# Identification of serum MicroRNAs associated with hepatic immunoinflammatory injury in chronic hepatitis B: implications for non-invasive diagnosis

**DOI:** 10.3389/fimmu.2025.1671149

**Published:** 2025-12-11

**Authors:** Mengkui Han, Junchi Xu, Xunxun Wu, Zhiqing Hu, Xiaoyuan Hu, Xiaolong Zhu, Cailin Chen, Qinkao Xuan, Yang Li, Chuanwu Zhu, Li Zhu, Xiaohua Yang, Jin Li

**Affiliations:** 1 Department of Infectious Diseases, The Affiliated Infectious Diseases Hospital of Soochow University, Suzhou, Jiangsu, China; 2 Department of General Surgery, The Affiliated Hospital of Jiangnan University, Wuxi, Jiangsu, China; 3 Department of General Surgery, The First Affiliated Hospital of Soochow University, Suzhou, Jiangsu, China; 4 Department of Gastroenterology, Jiangsu Shengze Hospital, Suzhou, Jiangsu, China

**Keywords:** biomarkers, CHB, HBV, HCC, liver fibrosis, miRNAs

## Abstract

**Background and aims:**

Hepatitis B virus (HBV) causes chronic hepatic infection, leading to various advanced liver diseases. Currently, there is still a lack of effective non-invasive biomarkers for evaluating hepatic inflammation and fibrosis. Circulating microRNAs (miRNAs) serve as key regulators and potential biomarkers in the progression and pathogenesis of HBV-associated progressive liver diseases. Here, we characterized the miRNA profile in chronic hepatitis B (CHB) patients and probed their association with liver inflammation and fibrosis.

**Approaches and results:**

We profiled 34 candidate miRNAs in serum from a well-characterized HBV Disease Continuum Cohort, comprising 165 individuals in the discovery set (42 healthy controls, HC; 40 CHB; 42 cirrhosis; 41 hepatocellular carcinomas, HCC) and 143 in an independent testing set. Serum miRNA levels were quantified by qRT-PCR. Supervised clustering and correlation analyses revealed distinct miRNA expression profiles across disease stages. Statistical analyses included logistic regression, and ROC/AUC evaluation with 5-fold cross-validation and external validation. Unsupervised clustering and correlation with histological G/S staging revealed stage-specific miRNA signatures: miR-224-5p, miR-125a-5p, and miR-15a-5p peaked in cirrhosis and strongly correlated with fibrosis stage (miR-224-5p: r = 0.606, p = 1.5E-12), while miR-200a-3p and miR-939-5p were predominantly upregulated in HCC. Critically, miR-224-5p emerged as a robust non-invasive biomarker for cirrhosis, with exceptional diagnostic accuracy (AUC = 0.973 in discovery; 0.906 in external validation), significantly outperforming APRI (0.803) and FIB-4 (0.809), and remained the sole independent predictor in multivariable analysis (p = 0.009). For HCC detection, the miR-200a-3p/AFP combined model achieved outstanding performance (AUC > 0.9), substantially improving upon AFP alone (0.737). Bioinformatic prediction of targets (297 for miR-224-5p; 616 for miR-200a-3p) highlighted associated in cancer- and senescence-related pathways; however, these associations are in silico and require experimental validation.

**Conclusion:**

We identify miR-224-5p as a fibroinflammatory activity indicator for early cirrhosis detection and miR-200a-3p as a synergistic enhancer of AFP for non-invasive HCC diagnosis, establishing a dual miRNA signature that spans the HBV disease continuum and addresses critical gaps in current risk stratification. These findings highlight the potential of specific serum miRNAs as non-invasive biomarkers for monitoring disease progression and improving the differential diagnosis during the process of HBV-related liver diseases.

## Introduction

Hepatitis B virus (HBV) is the most common cause of viral hepatitis, which is a leading cause of end-stage liver disease worldwide (1). WHO estimates that there are 1.2 million new infections each year. In 2022, 254 million people were living with chronic hepatitis B (CHB) infection, resulting in an estimated 1.1 million deaths, mostly from cirrhosis and hepatocellular carcinoma (HCC) (WHO, 2024). More than two-thirds of cirrhosis and HCC cases related to CHB are observed in the WHO African, South-East Asian and Western Pacific regions (2). HBV is a non-cytopathic virus, with immunopathological mechanisms predominantly mediating liver damage. Thus, both innate and adaptive immune responses are critical for controlling HBV infection, they also drive liver inflammation, leading to fibrosis, cirrhosis, and HCC (3). HBV vaccination has significantly reduced new infections and childhood transmission globally. While disparities in socioeconomic status result in uneven declines in HBV prevalence, with some regions still experiencing increasing rates. This highlights the challenge of eradicating HBV through vaccination alone (4). Functional cure of CHB - or hepatitis B surface antigen (HBsAg) loss after 24 weeks of therapy - is now the goal of treatment, but is rarely achieved with current therapy (5). Although universal treatment of HBV infection has been proposed (6), in clinical practice, the majority of patients initiate antiviral therapy only when liver damage is apparent. Early identification and intervention in CHB and cirrhosis can, to some extent, slow the progression of liver disease and even partially reverse fibrosis (7). While liver biopsy remains the gold standard for assessing early intrahepatic inflammation and fibrosis, its invasiveness and risk of complications limit its widespread use in clinical practice (8).

In terms of hepatic inflammation biomarkers, serum alanine transaminase (ALT) remains the most widely used marker due to its availability. While there is debate over the upper limit of normal (ULN), 40 IU/L is the most commonly used cutoff. Notably, about 30% of CHB patients with “normal” ALT have significant liver inflammation, which could progress without timely treatment (9, 10). In the field of liver fibrosis diagnostics, transient elastography, ultrasonography, and biomarkers like APRI or FIB-4 are used to assess liver disease, they often exhibit limited diagnostic performance for mild-to-moderate fibrosis (11, 12). Monocyte distribution width (MDW), a routine blood parameter reflecting heterogeneity among monocytes, has been reported to exhibit significant diagnostic value in differentiating CHB, liver cirrhosis (LC), and HCC. As a marker associated with innate immune activation, MDW may be influenced by various inflammatory and immunological processes (13). Serum ATP contributes to diagnosing CHB and predicting its poor clinical outcome, including LC and HCC (14). Although numerous non-invasive biomarkers have been explored, their performance, especially in early disease and across the CHB-cirrhosis-HCC continuum, remains inadequate. Liver biopsy with histological grading (G) and staging (S) remains the gold standard for assessing disease progression; yet most biomarker studies lack rigorous correlation with G/S scores. Despite advances in understanding the pathogenesis and management of chronic liver diseases, effective biomarkers for predicting and preventing liver disease progression remain limited.

MicroRNAs (miRNAs) are single-stranded non-coding RNA molecules, typically 19–25 nucleotides in length, that regulate gene expression post-transcriptionally (15). Since their discovery in serum and plasma, circulating miRNAs have emerged as promising biomarkers for various diseases (16, 17). They offer several advantages, including high stability under extreme conditions, sequence conservation, spatiotemporal expression specificity, and ease of detection. Despite the presence of ribonucleases in blood and other body fluids, circulating miRNAs remain remarkably stable, likely due to their encapsulation in lipid vesicles, association with RNA-binding proteins, or a combination of both (18–20). The significance of miRNAs in molecular biology was further underscored in 2024, when the Nobel Prize in Physiology or Medicine was awarded to Victor Ambros and Gary Ruvkun for their pioneering discovery of miRNAs and their fundamental contributions to elucidating the mechanisms of post-transcriptional gene regulation mediated by these small RNAs (21).

Accumulating studies have demonstrated that miRNAs are critically involved in the biological processes of HBV, including viral replication, transcription, and hepatocyte invasion, exerting either promotive or inhibitory effects on disease progression (22–26). As biomarkers, numerous miRNAs have shown potential as diagnostic and prognostic indicators for liver fibrosis and HCC (27–30). Our group found that lower baseline levels of serum miRNAs and HBsAg-carried miRNAs (let-7f, miR-22, miR-30a and miR-122) associated with YIC (antigen-antibody immunogenic complex based therapeutic vaccine) treatment response and the variation trend of these 4 miRNAs could have a prognostic value for responsiveness to YIC treatment (31). Besides, we suggested that a simplified scoring model composed of miR-210, miR-22 and ALT can reproducibly predict the sustained virological response of IFN-α therapy in CHB patients (20). Furthermore, our findings demonstrate that HBsAg-miR-939-IL-8 axis may play a crucial role in HBV-induced hepatic necro-inflammation and progression of advanced liver diseases (32). Taken together, our group has accumulated some experience in identifying miRNAs as biomarkers for HBV-related liver diseases and elucidating their roles in the progression of chronic HBV infection.

Building on our previous research and to address current challenges, we selected 34 candidate miRNAs associated with HBV infection, involving HBV replication, immune regulation, liver-enriched expression, and hepatocarcinogenesis. We investigated the circulating expression patterns of all candidate miRNAs across the stages of chronic HBV-related liver disease and performed correlation analyses with clinical parameters. Our aim is to identify potential biomarkers for early diagnosis and longitudinal monitoring of disease progression in HBV-related end-stage liver diseases.

## Material and methods

### Study population

A total of 308 participants were enrolled in this study, divided into a discovery cohort and an independent testing cohort, all recruited from the First Affiliated Hospital of Soochow University and the Fifth People’s Hospital of Suzhou between Oct 2021-Sep 2025. The discovery cohort consisted of 165 individuals: 40 patients with CHB, 42 patients with HBV-related cirrhosis, 41 patients with HBV-related HCC, and 42 healthy controls (HC). The independent testing cohort consisted of 143 additional participants, with matched disease spectrum and identical enrollment criteria: 35 patients with CHB, 33 patients with HBV-related cirrhosis, 35 patients with HBV-related HCC, and 40 HC.

The following inclusion criteria were used: (1) CHB, positivity for HBsAg for longer than 6 months and viral loads over 10^2^ copies per milliliter (33); (2) HBV-related cirrhosis, diagnosed by two experienced pathologists via liver biopsy (34); HBsAg and HBV DNA positivity; (3) HBV-related HCC, diagnosed by two experienced pathologists via liver biopsy or hepatic resection surgery; HBsAg or HBV DNA positivity (35). Further exclusion criteria included: (1) liver damage induced by co-infection with hepatitis A, C, E virus; (2) evidence of liver comorbidities consequent to alcohol, drugs and autoimmune disease; (3) serious concurrent medical illnesses, including other malignant tumors, severe cardiopulmonary disease, psychiatric disorders and uncontrolled diabetes mellitus; (4) pregnancy or nursing. The level of fibrosis and inflammation was assessed using the Modified Gansheng Histological Scoring System (G/S staging) (36, 37). Eighty-two healthy controls were recruited from the department of physical examination of The Fifth People’s Hospital of Suzhou. All of them had normal ALT values (<40 U/L) and were tested negative for hepatitis B, C virus and HIV. This study was approved by the Ethics Committee of The Affiliated Infectious Diseases Hospital of Soochow University (Approval No. SZWY2023007) and conducted in accordance with the Declaration of Helsinki. Written informed consent was obtained from all participants.

### Candidate miRNAs screening

According to our previous studies, the miRNAs database and the published literatures, thirty-four miRNAs previously reported to be dysregulated in HBV infection or associated with disease progression were included into this study for screening, specifically in regulation of HBV replication, modulation of HBV-induced immune responses, liver-enriched expression, and HBV-driven hepatocarcinogenesis. All miRNA-specific stem-loop reverse transcription primers and forward qPCR primers were designed and synthesized by RiboBio Co., Ltd. (Guangzhou, China) and GeneAdv Co., Ltd. (Suzhou, China); the corresponding primer sequences are listed in **Supplementary Table 1**.

### Total RNA isolation

Prior to RNA extraction, serum samples underwent sequential centrifugation at 2,000 g for 20 min, then 10,000 g for 30 min, followed by 12,000 g for 10 min to completely remove cellular debris. Total RNA was extracted from 100 μL of serum using TRIzol^®^ LS reagent (Invitrogen, USA) according to the manufacturer’s instructions. Synthetic Caenorhabditis elegans miRNA cel-miR-39 (QIAGEN, Germany) was spiked in as an external reference.

### MiRNA reverse transcription and qRT-PCR

The extracted total RNA was conducted miRNA reverse transcription using Multiscribe reverse transcriptase (Thermo Fisher Scientific-Applied Biosystems, Waltham, MA, USA) on RT reaction (16 °C, 30 min; 42 °C, 1 h; 85 °C, 5 min). Real-time PCR reactions were performed using 2 × Universal SYBR Green Fast qPCR Mix (ABclonal, China) in the Applied Biosystems 7500 Real-Time PCR System (Thermo Fisher Scientific, USA). The reactions, running in duplicate, were carried out in 96-well plates at 95 °C for 1 min, followed by 40 cycles of 95 °C for 10 s and 60 °C for 30 s. Following 40 cycles of amplification, melting curve analysis was performed in each run. Melting curve analysis was conducted after amplification. Expression levels were normalized to cel-miR-39 and calculated using the 2^−ΔCt^ method.

### Statistical analysis

Statistical analyses were performed using SPSS version 26.0 (IBM Corp.). Continuous variables are presented as median (interquartile range). Parametric tests (ANOVA) and non-parametric tests (Mann-Whitney U or Kruskal-Wallis test) were applied based on data distribution. Differences in miRNA expression across the four groups were assessed by Dunn’s multiple comparisons test for multiple pairwise comparisons. Categorical variables were analyzed using Pearson chi-square test, continuity correction, or Fisher’s exact test as appropriate. Correlation analysis was conducted using Spearman correlation. Univariate and forward stepwise multivariate logistic regression identified independent predictors. Binary logistic regression and ROC curve analyses were used to assess the diagnostic performance of individual and combined miRNAs for differentiating CHB, cirrhosis, and HCC. The optimal cutoff value was determined by maximizing the Youden Index. A p-value < 0.05 was considered statistically significant. ****, p<0.0001; ***, p<0.001; **, p<0.01; *, p<0.05; ns, p≥0.05.

## Result

### Clinical characterization of the HBV disease continuum cohort

The study cohort comprised four clinically defined groups: HC, CHB, cirrhosis, and HCC, with the HC group serving as the reference control. No statistically significant differences were observed in age or gender distribution among the four groups (p > 0.05, **Table 1**). However, significant differences in HBsAg levels were detected among the CHB, cirrhosis, and HCC groups (p = 2.3E-06, **Table 1**), with the HCC group exhibiting markedly lower HBsAg levels compared to the other two groups. Similarly, HBV DNA levels differed significantly among these three groups (p = 0.0004, **Table 1**), primarily due to the higher viral loads observed in the CHB group. The ALT and AST levels were elevated in all HBV-infected groups compared to the HC group, with no or little significant differences observed among the three HBV-infected groups (CHB, cirrhosis, and HCC) (**Table 1**, **Supplementary Figure 1AB**). Based on the histological grading and staging criteria (G/S classification), the majority of CHB patients were classified as G2, reflecting mild inflammatory activity. Cirrhosis patients were predominantly distributed across stages G2 - G4, whereas HCC patients were primarily categorized within grades G1 - G4. Notably, the liver inflammation grade was significantly higher in the cirrhosis group compared to both the CHB and HCC groups (**Table 1**, **Supplementary Figure 1C**). In terms of fibrosis staging, the majority of CHB patients were classified as S2, indicative of mild fibrosis, while cirrhosis patients were all categorized as S4, representing advanced fibrosis. HCC patients exhibited a broader range of fibrosis stages from S2 to S4, with cirrhosis patients demonstrating the highest fibrosis staging among all HBV-infected groups (**Table 1**, **Supplementary Figure 1D**). AFP levels exhibited a gradual increase across the four groups, with the highest levels detected in the HCC group. Significant differences in AFP levels were noted between the HCC group and each of the other groups (**Table 1**, **Supplementary Figure 1E**). Additional data on other liver function parameters, including albumin (ALB), total bilirubin (TBil), and alkaline phosphatase (ALP) are also provided (**Table 1**, **Supplementary Figures 1F–H**).

**Table 1:** Basic characteristics of enrolled patients at baseline.

Variables	HC (n=42)	CHB (n=40)	Cirrhosis (n=42)	HCC (n=41)	P Value
Age (year)	49.0 (40.8-63.0)	47.5 (41.5-55.8)	49.5 (43.8-57.0)	55.0 (48.0-60.0)	0.0634^a^
Gender (Male/Female)	29/13	27/13	30/12	33/8	0.5601^b^
HBsAg (log_10_IU/ml)	–	3.2 (2.4-3.7)	3.1 (2.5-3.6)	2.5 (1.8-3.3)	2.3E-06^a^
HBV DNA (log_10_copies/ml)	–	4.8 (3.7-6.8)	2.7 (2.7-4.7)	3.3 (2.5-4.8)	0.0004^a^
ALT (U/L)	14.5 (10.0-19.3)	31.0 (19.3/54.3)	31.0 (19.0-41.0)	34.9 (21.2-52.8)	0.5240^c^
AST (U/L)	19.0 (16.8-22.3)	27.0 (20.3-34.8)	31.5 (25.0-44.0)	32.0 (23.9-72.4)	0.0302^c^
G (0/1/2/3/4)	–	0/2/36/2/0	0/0/10/24/8	0/5/17/15/4	3.5E-06^b^
S (0/1/2/3/4)	–	0/2/30/8/0	0/0/0//42	0/3/14/12/12	1.8E-11^b^
AFP (μg/L)	2.8 (2.2-3.3)	3.3 (1.9-4.9)	4.9 (2.9-10.4)	84.3 (3.6-1712.0)	7E-07^c^
TBil (μmol/L)	9.3 (7.7-12.6)	14.6 (10.9-16.9)	18.7 (13.0-32.6)	19.7 (14.5-29.3)	0.0002^c^
AlB (g/L)	45.1 (44.2-47.1)	44.1 (41.1-48.1)	40.3 (33.6-46.4)	39.7 (38.2-42.7)	0.0002^c^
ALP (U/L)	77.0 (67.0-93.3)	79.5 (67.5-101.5)	85.5 (68.5-119.5)	84.3 (65.5-102.2)	0.3916^c^

Data are median (25% percentile – 75% percentile) except gender and G\S stage. Abbreviations: HC, Healthy control; CHB, chronic hepatitis B; Cirrhosis, HBV with cirrhosis patients; HCC, HBV with hepatocellular carcinoma patients; HBsAg, surface antigen of hepatitis B virus; HBV DNA, hepatitis B virus deoxyribonucleic acid; TBil, total bilirubin; ALT, alanine aminotransferase; AST, aspartate aminotransferase; AFP, alpha fetoprotein; ALB, albumin; ALP, alkaline phosphatase; G, inflammation stage; S, fibrosis stage. a: Kruskal-Walli’s test; b: Chi-square test; c, an ordinary one-way ANOVA test was performed, with the comparison limited to the CHB, Cirrhosis, and HCC groups.

### Global miRNA expression profiles in the HBV disease continuum cohort

To investigate the miRNA expression profiles in individuals with HBV disease continuum cohort, 34 candidate miRNAs were selected based on our previous findings and recent literature (**Supplementary Table 1**). The expression levels of these miRNAs were quantified in serum samples from 165 enrolled individuals in the cohort of HBV Disease Continuum (HC, CHB, cirrhosis, HCC). Initially, the CHB, cirrhosis, and HCC groups were collectively classified as the HBV-infected group (n = 123), while the HC group served as the reference cohort (n = 42). The fold change of each miRNA in the HBV-infected group relative to the HC group was then calculated. Among the 34 candidate miRNAs, 17 miRNAs exhibited at least a 2-fold upregulation in the HBV-infected group compared to the HC group. Notably, the levels of miR-224-5p (fold change = 16.012, p = 1.1E-20), miR-15a-5p (fold change = 6.122, p = 0.0090), and miR-125a-5p (fold change = 4.949, p = 7.84E-05) were significantly elevated in the HBV-infected group compared to the HC group, with fold changes exceeding 4-fold (p < 0.01, **Supplementary Table 2**). Moreover, miR-145-5p was found to be the most significantly downregulated in the HBV-infected group, with a fold change of 0.265 (p=3.59E-14). To further analyze the distribution profiles of the 34 miRNAs across the four groups, we compared the expression levels of 34 miRNAs among HC, CHB, cirrhosis and HCC groups. It was found that miR-224-5p displayed the most significant difference among these four groups (p = 6.39E-27). Besides, miR-125a-5p (p =4.95E-16), miR-145-5p (p =3.15E-15), miR-15a-5p (p = 9.05E-11) also showed significant difference among the four groups (**Supplementary Table 3**).

### Stage-specific circulating miRNA signatures across the HBV disease continuum

**Figure 1:**
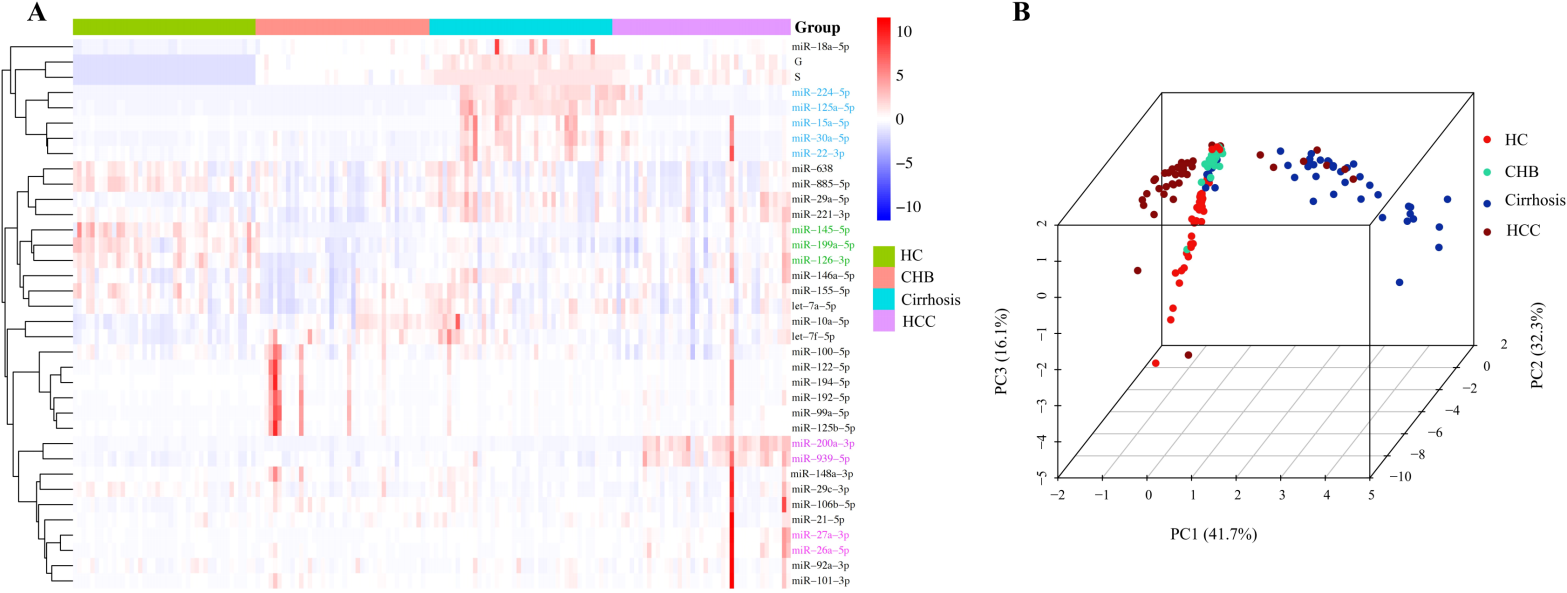
Unsupervised Clustering and Principal Component Analysis (PCA) of Plasma miRNA Expression Profiles across HC, CHB, Cirrhosis, and HCC Groups: **(A)** Heatmap with hierarchical clustering of 30 differentially expressed plasma miRNAs (rows) across all study samples (columns). Color intensity reflects relative expression levels (2^-^ΔCt), with red indicating upregulation and blue indicating downregulation. Samples are grouped by clinical status: healthy controls (HC, green), chronic hepatitis B (CHB, red), cirrhosis (cyan), and hepatocellular carcinoma (HCC, purple). Clustering reveals distinct expression patterns associated with disease progression. **(B)** Three-dimensional PCA plot based on the top 3 principal components (PC1: 41.7%, PC2: 32.3%, PC3: 16.1%), showing clear separation between HC, CHB, cirrhosis, and HCC groups. Each dot represents an individual sample, colored by group. The results demonstrate that global miRNA expression profiles can effectively distinguish disease stages, supporting their potential as molecular classifiers for liver disease progression. HC, Healthy control; CHB, chronic hepatitis B; Cirrhosis, HBV with cirrhosis patients; HCC, HBV with hepatocellular carcinoma patients.

In order to explore the association between miRNA expression profiles and disease progression of HBV disease continuum, a supervised clustering analysis incorporating the 34 candidate miRNAs and histological G/S staging was conducted to visualize global miRNA expression profiles across the four groups, including HC, CHB, cirrhosis and HCC. The data revealed that miR-224-5p, miR-125a-5p, miR-15a-5p, miR-30a-5p, and miR-22-3p were closely clustered and showed similar trend with G/S staging, exhibiting high expression in cirrhosis group (Blue font, **Figure 1A**). On the other hand, miR-200a-3p and miR-939-5p were predominantly upregulated in the HCC group. Additionally, miR-27a-3p and miR-26a-5p also showed the similar trend with the two aforementioned miRNAs, while with a weak increasing trend in HCC group (Purple font, **Figure 1A**). Additionally, miR-145-5p, miR-199a-5p and miR-126-3p were closely clustered because of their higher expression levels in HC group (Green font, **Figure 1A**).

Taken together, several miRNAs exhibited distinct expression patterns across the HBV disease continuum. Specifically, miR-224-5p, miR-125a-5p, and miR-15a-5p demonstrated the highest expression levels in the cirrhosis group. In contrast, miR-200a-3p and miR-939-5p were predominantly upregulated in the HCC group. Notably, miR-145-5p showed relatively higher expression levels in the HC group. Therefore, the 3D principal components analysis was conducted to distinguish (similarity versus difference) between various patient groups. It was found that miR-224-5p, miR-125a-5p, miR-15a-5p, miR-200a-3p, miR-939-5p combined with miR-145-5p can effectively distinguish the four groups. We observed that cirrhosis group (dark blue points) and HCC group (brown points) formed independent clusters, respectively. And, CHB patients (green points) showed completely separation from cirrhosis and HCC groups, while HC group (red points) appeared as a distinct subset (**Figure 1B**).

### MiR-224-5p strongly correlates with histological G/S grading and enables high-accuracy detection of cirrhosis

**Figure 2:**
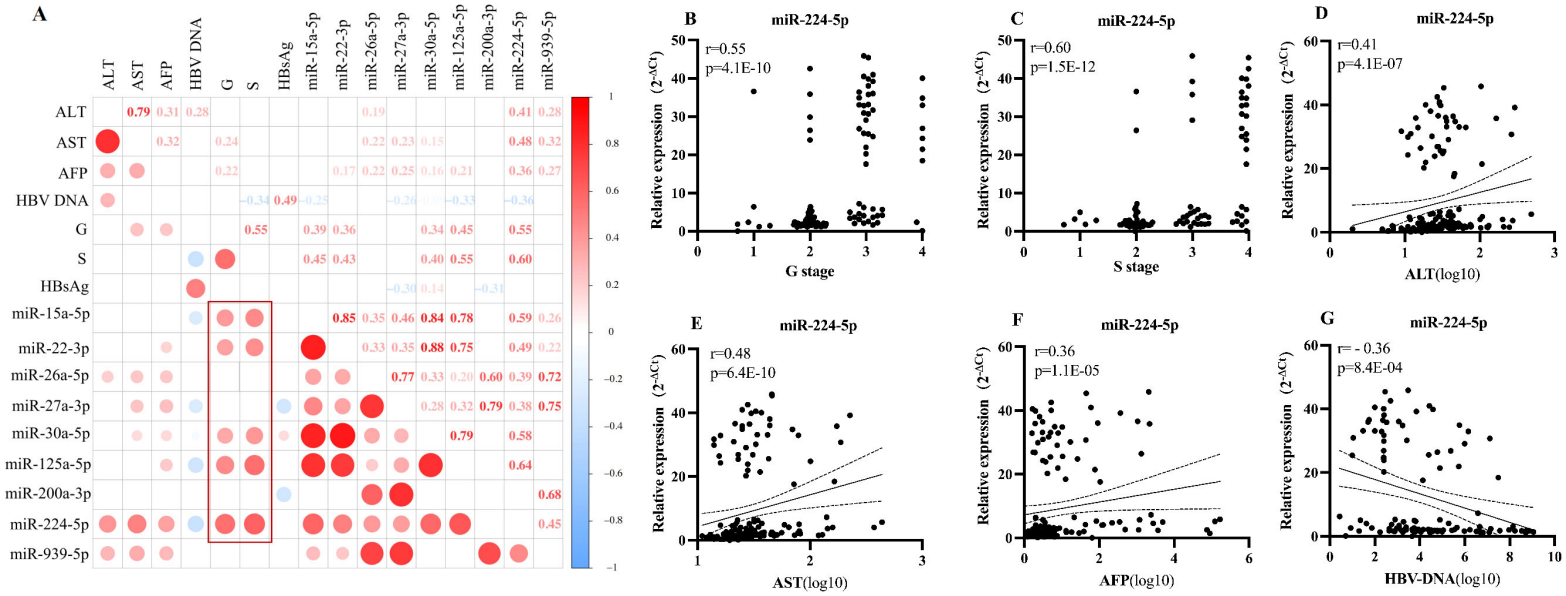
Correlation Analysis between miR-224-5p and Clinical Parameters in HBV-Infected Patients. **(A)** Correlation matrix analysis of highly expressed miRNAs with pathological grading of liver inflammation/fibrosis, key inflammation markers, and virological variables in cirrhosis and hepatocellular carcinoma cohorts. The significant differences in miR-224-5p were analyzed in conjunction with clinical and Enrichment analysis: **(B, C)** The analysis results of the correlation between miR-224-5p and G/S staging; **(D–G)** Correlation analysis of relative miR-224-5p expression levels with clinical indicators in a cohort of HBV-infected patients.

The significant clustering of these miRNAs suggests a similar expression pattern among them and indicates a clear association with the progression of HBV disease continuum. Among the four groups, miR-224-5p, miR-125a-5p, miR-15a-5p, miR-22-3p, and miR-30a-5p showed highest levels in cirrhosis group, with significant difference between cirrhosis vs CHB, cirrhosis vs HC and cirrhosis vs HCC. Importantly, miR-224-5p displayed more differentially expression levels among the four groups. MiR-224-5p expressed higher in CHB than in HC, higher in cirrhosis than in CHB and HC, and also higher in HCC than in CHB and HC (**Supplementary Figure 2**, **Supplementary Table 4**). The data suggested that the expression trend of miR-224 was more similar to G/S grading. The correlation matrix analysis, which was conducted in CHB, cirrhosis and HCC groups, showed that miR-224-5p (r=0.55, r=0.60), miR-125a-5p (r=0.45, r=0.55), miR-15a-5p (r=0.39, r=0.45), miR-22-3p (r=0.36, r=0.43), miR-30a-5p (r=0.34, r=0.40) and were significantly correlated with histopathological staging (G/S staging) in HBV infected patients (**Figure 2A**, n=123). Collectively, among the 34 candidate miRNAs, miR-224-5p displayed the most significant clustering and positive correlation with G/S grading in the HBV disease continuum cohort. Focused on miR-224-5p, we found that the levels of miR-224-5p not only displayed most significantly positive correlation with GS grading (G: r=0.55, p=4.1E-10; S: r=0.60, p=1.5E-12) (**Figures 2B, C**), but also showed significant positive correlations with ALT, AST, and AFP (ALT: r = 0.41, p=4.1E-07; AST: r = 0.48, p=6.4E-10;AFP: r = 0.36, p=1.1E-05). In contrast, miR-224-5p expression exhibited a significantly negative correlation with HBV DNA levels (r =–0.36, p = 8.4E-04) (**Figures 2D–G**).

**Figure 3:**
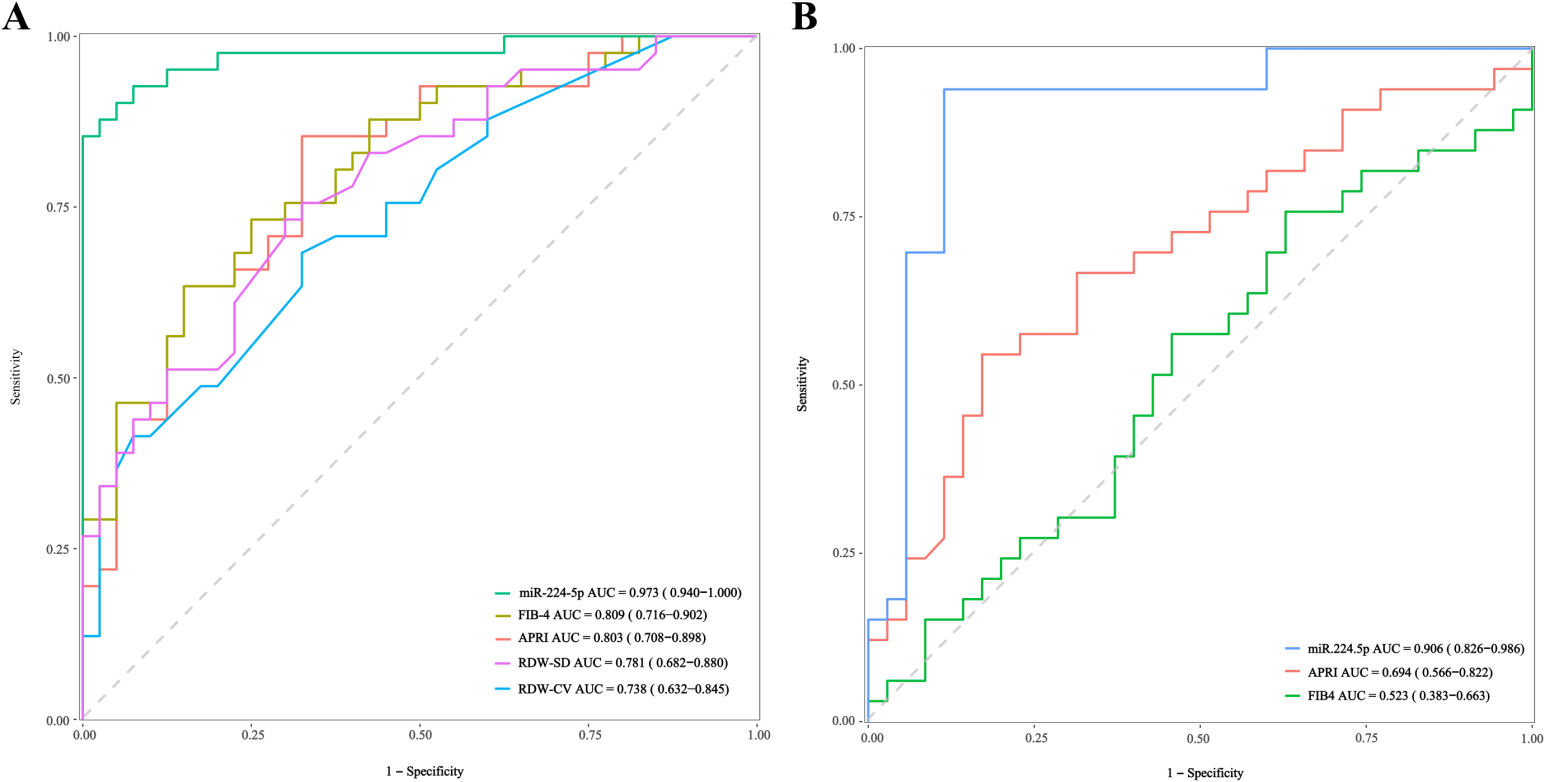
Diagnostic Performance of miR-224-5p and Conventional Biomarkers in the Training Set **(A)** and External Validation Cohort **(B)**: **(A)** Receiver operating characteristic (ROC) curves for miR-224-5p, FIB-4, APRI, RDW-SD, and RDW-CV in the training set. The area under the curve (AUC) with 95% confidence interval (CI) is shown for each marker. **(B)** ROC curves for the same biomarkers in an independent external validation cohort.

Given the high expression of miR-224 in cirrhosis group and its significant positive correlation with G/S grading, we speculated that miR-224 may have potential diagnostic value in differentiating cirrhosis patients from CHB patients. Univariate and multivariate analyses revealed that, among the five miRNAs significantly upregulated in cirrhosis group, only miR-224-5p remained significantly associated with cirrhosis in the multivariable analysis (p=0.009, **Supplementary Table 5**). We further performed ROC curve analysis to compare the diagnostic performance of serum miR-224-5p with that of conventional non-invasive fibrosis biomarkers. The ROC data showed that miR-224-5p exhibited the highest diagnostic accuracy (AUC = 0.973), markedly exceeding that of FIB-4 (AUC = 0.809), APRI (AUC = 0.803), Red Cell Distribution Width – Standard Deviation (RDW-SD) (AUC = 0.781) and Red Cell Distribution Width – Coefficient of Variation (RDW-CV) (AUC = 0.738) for identifying cirrhosis (**Figure 3A**, **Supplementary Table 6**). To assess the robustness and generalizability of miR-224-5p as a diagnostic biomarker for differentiating HBV-related cirrhosis from CHB patients, we performed 5-fold cross-validation within the discovery cohort. In the internal training set validation, miR-224-5p achieved near-perfect discrimination, with individual fold AUCs ranging from 0.861 to 1.000 and a mean AUC of 0.950 (**Supplementary Figure 3A**). In the internal validation set, the classifier maintained high discriminatory power, with a mean AUC of 0.917 and individual fold AUCs between 0.833 and 1.000 (**Supplementary Figure 3B**). To further evaluate the generalizability of miR-224-5p, we validated its diagnostic performance in an independent external cohort (**Supplementary Table 7**). Consistently, serum miR-224-5p maintained high predictive accuracy for identifying cirrhosis, achieving an AUC of 0.906 (**Figure 3B**). The consistency of high AUC values across both discovery and testing cohorts suggests that miR-224-5p is not overfitted and possesses strong predictive capacity for the identification of cirrhosis in CHB patients.

**Figure 4:**
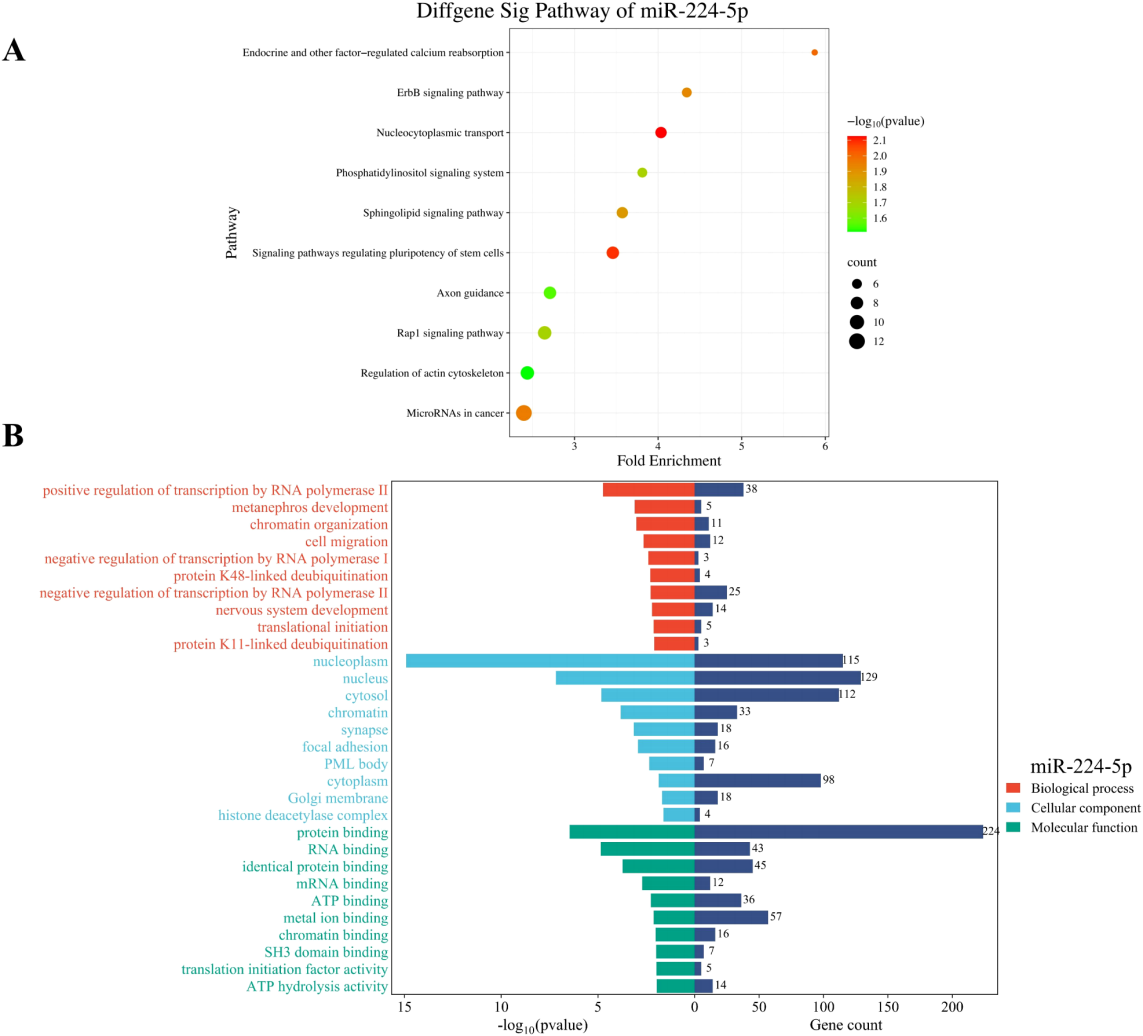
Functional enrichment analysis of target genes regulated by miR-224-5p: **(A)** The top 10 KEGG pathways. **(B)** The 10 top enriched GO terms. BP, biological process; CC, cellular component; MF, molecular function. The P values of GO terms and KEGG pathways decrease from top to bottom.

To explore the functional implications of miR-224-5p, which was markedly upregulated in cirrhosis and significantly correlated with GS grading, target gene prediction was conducted using established online miRNA target databases (Targetscan, miRmap, mirDIP). A total of 297 putative target genes were identified for miR-224-5p. Functional enrichment analysis based on the KEGG pathway database suggested that these 297 putative target genes predominantly associated with the pathways such as “microRNAs in cancer,” “nucleocytoplasmic transport,” and “signaling pathways regulating pluripotency of stem cells”, which still require experimental validation (**Figures 4A, B**).

### Identification of a novel miRNA signature for early detection of HBV-related HCC

Building upon the findings from the clustering analysis, a distinct expression pattern was observed for four microRNAs among HCC subgroups. Specifically, the circulating levels of miR-26a-5p, miR-27a-3p, miR-200a-3p and miR-939-5p were significantly higher in HCC patients compared to those in the HC, CHB, and cirrhosis groups (**Figure 1**). Among the four groups, miR-26a-5p, miR-27a-3p, miR-200a-3p and miR-939-5p showed higher levels in HCC compared with cirrhosis, CHB and HC, respectively. Besides, miR-939-5p also displayed higher levels in cirrhosis than in CHB groups (**Supplementary Figure 4**, **Supplementary Table 4**).

**Figure 5:**
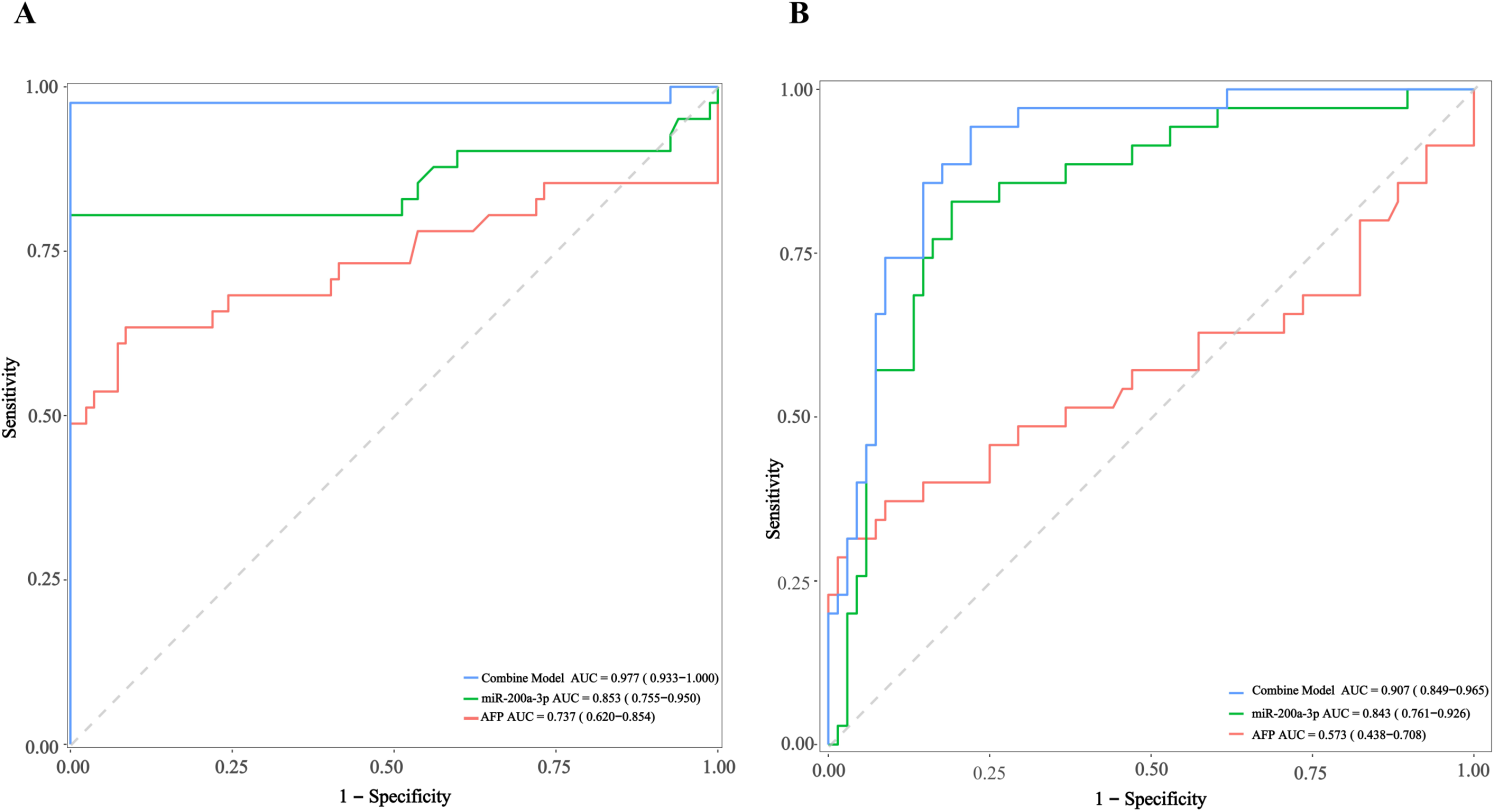
Diagnostic performance of the combined model, miR-200a-3p, and AFP for predicting HCC in patients with chronic hepatitis B or cirrhosis — training set **(A)** and External Validation Cohort **(B)**. **(A)** Receiver operating characteristic (ROC) curves for the combined model (integrating miR-200a-3p + AFP), miR-200a-3p alone, and AFP alone in the training set. **(B)** ROC curves for the same models in an independent external validation cohort. The combined model maintained excellent performance, demonstrating strong generalizability.

Further, to enhance clinical applicability, particularly for distinguishing HCC from non-HCC chronic liver disease, we combined the CHB and cirrhosis groups into a single “CHB/cirrhosis” group. Univariate and multivariate analyses revealed that, among the four miRNAs significantly upregulated in cirrhosis group, only miR-200a-3p significantly associated with cirrhosis in the multivariable analysis (p=3.36E-04, **Supplementary Table 8**). Thus, for the differential diagnosis between CHB/Cirrhosis and HCC, ROC curves were constructed to assess the predictive potential of miR-200a-3p and AFP. The AUC values showed that the miR-200a-3p (AUC=0.853) showed higher prediction value than AFP (AUC=0.737) (**Figure 5A**, **Supplementary Table 9**). To further improve diagnostic performance of this miRNAs, Binary logistic regression analysis was conducted. Binary logistic regression analysis led to the combination of miR-200a-3p and AFP as independent constituents for the predictive model, logit(p) = 51.073 - 1.866 * miR-200a-3p + 0.002 * AFP. The AUC, as determined by ROC curve analysis, yielded a value of 0.977 in the discovery cohort (**Figure 5A**, **Supplementary Table 9**). To evaluate the reproducibility of miR-200a-3p as an HCC-specific biomarker, we applied 5-fold cross-validation in the discovery cohort. While performance in the training folds was modest (mean AUC = 0.776; range: 0.600 - 1.000, **Supplementary Figure 5A**), the held-out validation folds yielded robust discrimination (mean AUC = 0.889; range: 0.800 -1.000; **Supplementary Figure 5B**), suggesting model stability despite inter-fold variability. Critically, in the independent external cohort, the miR-200a-3p/AFP combined model achieved excellent diagnostic accuracy for HCC detection (AUC = 0.907; **Figure 5B**), markedly outperforming either marker alone. This consistent high performance across internal and external validation underscores the potential of miR-200a-3p, not as a standalone test, but as a synergistic enhancer of AFP, for non-invasive identification of HCC in high-risk patients with underlying CHB or cirrhosis.

**Figure 6:**
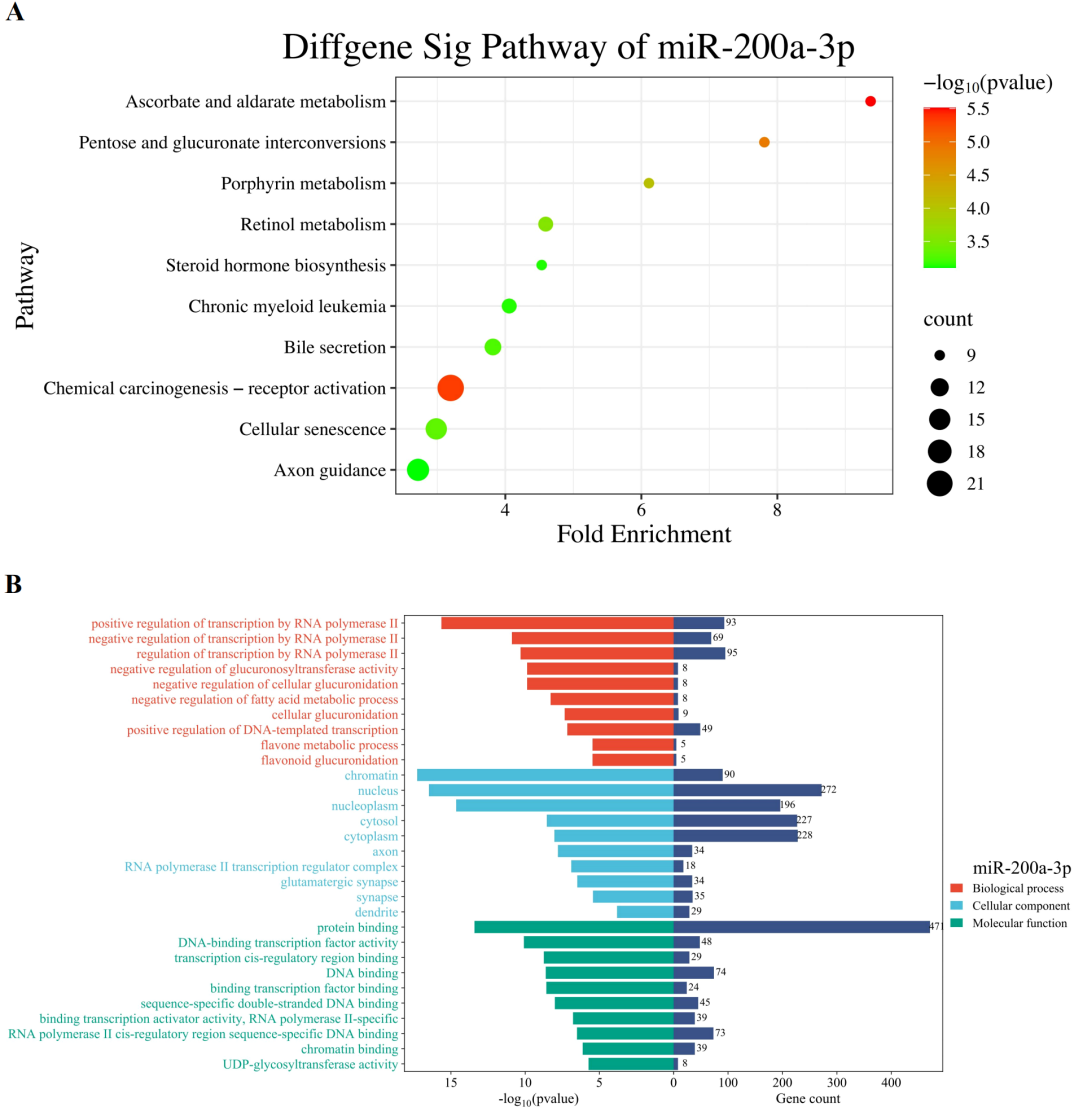
Enrichment analysis of target genes of DEMs miR-200-3p: **(A)** The top 10 KEGG pathways of miR-200-3p. **(B)** The 10 top enriched GO terms of miR-200-3p. BP, biological process; CC, cellular component; MF, molecular function. The P values of GO terms and KEGG pathways decrease from top to bottom.

Given the HCC-specific upregulation of miR-200a-3p, putative targets of miR-200a-3p were predicted using an integrated approach across the miRNA target databases (Targetscan, miRmap, mirDIP). A total of 616 putative target genes for miR-200a-3p were identified through in silico prediction. KEGG pathway enrichment analysis of these predicted targets revealed statistically significant overrepresentation in pathways, including “chemical carcinogenesis - receptor activation”, “cellular senescence”, and “ Transcriptional regulation” (**Figures 6A, B**). Importantly, this analysis is hypothesis-generating; the observed pathway links do not imply direct functional roles of miR-200a-3p in tumorigenesis or signal transduction and require rigorous experimental validation.

## Discussion

Chronic hepatitis B, caused by persistent HBV infection, progresses from an asymptomatic carrier state to liver fibrosis, cirrhosis, and ultimately HCC, representing a major global health burden and a leading cause of cancer-related mortality (38–40). The clinical outcome of chronic HBV infection is primarily determined by the dynamic interplay between viral factors and host immune responses. Notably, HBV is a non-cytopathic virus, and liver damage is predominantly mediated by the host immune response. During the course of HBV infection, the immune system plays a dual role: it contributes to viral clearance by targeting and eliminating infected hepatocytes, but concurrently drives hepatic inflammation and exacerbates liver injury (3). Hepatic inflammatory response is a key mediator in the pathogenesis of CHB and its progression towards cirrhosis and HCC (41, 42). Chronic hepatitis B is primarily managed through the use of oral nucleot(s)ide analogues, interferon therapies, or a combination of both (43). Despite these interventions, most patients fail to achieve complete viral eradication or sustained disease resolution (44). This limitation can largely be attributed to an incomplete understanding of the complex interplay between HBV and the host immune response during various stages of infection and disease progression. Notably, one potential mechanism of host-virus interaction involves HBV’s modulation of host miRNAs expression. By altering miRNA profiles, HBV may enhance its replication within hepatocytes and establish a favorable microenvironment that supports viral persistence and contributes to the development of liver disease (45, 46).

Our study focused on the multiple miRNAs’ profiles during the progress of HBV disease continuum, including CHB, cirrhosis and HCC, HC serving as controls. Based on our previous studies, in combination with the latest literature reports and miRNA databases, we have screened out 34 candidate miRNAs for further investigation. We have found that miR-224-5p, miR-125a-5p, miR-15a-5p, miR-30a-5p, and miR-22-3p were closely clustered and showed similar trend with G/S staging, exhibiting high expression in cirrhosis group. Besides, miR-200a-3p and miR-939-5p were predominantly upregulated in the HCC group, and miR-27a-3p and miR-26a-5p also showed a slight increase in HCC group.

It is worth noting that miR-224-5p exhibited the highest fold change (fold change = 16.012, p = 1.1E-20) when comparing the HC group with the HBV infection group (CHB + cirrhosis + HCC). Additionally, miR-224-5p showed the closest clustering with G/S grading, and correlation analysis also revealed that miR-224-5p expression displayed the most significantly positive correlation with G grade (r=0.55), S stage (r=0.60), ALT (r=0.41), AST (r=0.48), and AFP (r=0.36) levels, suggesting that miR-224-5p may play a critical role in hepatic inflammatory responses and the progression of advanced liver diseases. Among fibrosis-associated miRNAs, miR-224-5p emerged as the sole independent predictor of cirrhosis in multivariable analysis. Notably, it demonstrated exceptional and reproducible diagnostic accuracy for distinguishing cirrhosis from CHB, achieving AUCs of 0.973 (discovery), 0.950 (cross-validation), and 0.906 (independent validation), consistently outperforming conventional non-invasive indicators (APRI, FIB-4).

To date, the existing literature has reported that miR-224-5p is significantly upregulated in HCC. It was found that miRNA-224-5p expression level in HCC was higher than in non-tumor tissues. However, the circulating miR-224 levels, which are more favorable for early non-invasive clinical diagnosis, were not detected (47, 48). In terms of mechanism, it was already reported that E2F1−mediated upregulation of miR−224−5p may serve an important role in liver cancer cell migration, invasion, and epithelial−mesenchymal transition (EMT) by targeting melanoregulin (MREG), highlighting the critical regulatory role of miR−224−5p in liver cancer progression (49). However, these works relied on tissue-based, binary comparisons (HCC vs. non-tumor) and did not assess circulating miR-224-5p across the full HBV disease spectrum or correlate it with histological inflammation/fibrosis. In contrast, our study provides the first comprehensive serum profiling of miR-224-5p in a histologically discovery and testing cohorts spanning healthy controls, CHB, cirrhosis, and HCC. While we confirm its elevation in HCC versus HC and CHB, we reveal a novel pattern: miR-224-5p peaks in cirrhosis, significantly exceeding levels in both CHB and HCC. Critically, it shows the strongest correlation with G/S staging among all 34 miRNAs, reflecting active immune-mediated liver injury rather than malignancy per se. This aligns with the established model that HBV is non-cytopathic and that chronic immune-driven inflammation and fibrogenesis underlie progression to cirrhosis and HCC (3, 50). More importantly, our study focused on the circulating levels of miR-224-5p, which holds significant clinical implications for its use as a non-invasive biomarker. Thus, we reframe miR-224-5p not as a tumor-specific marker, but as a non-invasive indicator of fibroinflammatory activity in the pre-neoplastic stage. Our findings extend prior literature by placing miR-224-5p within a disease-continuum context, offering a more precise, stage-specific biomarker for monitoring HBV-related liver disease progression.

The study also found that the expression levels of miR-26a-5p, miR-27a-3p, miR-200a-3p, and miR-939-5p were significantly upregulated in the HCC group compared to the HC, CHB, and cirrhosis groups. More importantly, miR-200a-3p combined with AFP showed effective predictive performance in the differential diagnosis between CHB/cirrhosis and HCC (AUC > 0.9) in the discovery and testing cohorts. Besides, the predictive model consisting of miR-200a-3p and AFP also displayed significant predictive capability for the differential diagnosis of HCC in the cross validation. Our results demonstrated that miR-200a-3p not only highly expressed in HBV-infected associated HCC, but also significantly improves the diagnostic performance of AFP for HBV-HCC when used in combination.

MiR-200a-3p has been implicated in various malignancies, including gastric cancer, bladder cancer, and hepatocellular carcinoma, participating in multiple pathophysiological processes involved in tumor development (51, 52). In a cohort of 136 HCC patients undergoing trans-arterial chemoembolization (TACE), miR-224-5p was found to be significantly associated with overall survival in univariate analysis, suggesting a potential prognostic role. However, it did not remain an independent predictor in multivariate analysis. In contrast, miR-200a-3p emerged as a strong independent prognostic factor for survival (p<0.001), outperforming conventional clinical markers. It demonstrated high predictive accuracy (AUC = 0.853; sensitivity = 80.5%, specificity = 100.0%) and, when combined with AFP and satellite nodules, further improved prognostic performance (AUC = 0.977). These findings highlight serum miR-200a-3p as a promising biomarker to guide personalized TACE therapy in HCC (53). Notably, the independent prognostic value of serum miR-200a-3p in HBV-HCC patients, as reported by Liu et al. in a TACE cohort, aligns closely with our observation of its stage-specific upregulation in HCC. Interestingly, both our study and Liu et al. (2014) observed that while miR-224-5p is elevated in HCC, it fails to retain independent prognostic significance in multivariable models, unlike miR-200a-3p. This convergence suggests that miR-224-5p elevation primarily reflects the underlying fibroinflammatory liver disease burden rather than intrinsic tumor aggressiveness.

We fully agree that miR-224-5p alone is suboptimal for distinguishing cirrhosis from HCC, as it is elevated in both stages. Recognizing this limitation, we developed alternative, highly accurate diagnostic models: notably, the miR-200a-3p/AFP combination achieves an AUC > 0.9 for differentiating HCC from CHB/cirrhosis, fulfilling the need for precise HCC detection in high-risk cirrhotic patients. In summary, both our study and prior literature confirm that miR-224-5p is significantly elevated in HCC versus healthy individuals. Our unique contribution is demonstrating that miR-224-5p is most strongly associated with G/S staging, reaches its highest level in cirrhosis (where inflammatory and fibrotic activity is maximal), and thus serves as a robust non-invasive biomarker for distinguishing CHB from cirrhosis (AUC > 0.9). More importantly, this finding mechanistically links miR-224-5p to the immune-pathological processes driving HBV-related end-stage liver disease, offering new insight into its role in the inflammation-fibrosis-cirrhosis-HCC axis. While, we demonstrated that miR-200a-3p were significantly elevated in the serum of HBV-related HCC patients and exhibited good diagnostic performance in distinguishing HBV-related HCC from other conditions, highlighting its potential as biomarker for the early diagnosis of HBV-related HCC.

In summary, this study utilized qRT-PCR technology to screen for miRNAs specifically expressed in the cohort of HBV disease continuum. Analysis with clinical indicators revealed that differentially expressed miRNAs were associated with liver injury, inflammatory response, fibrosis level, and HCC occurrence. These findings provide valuable references for the study of HBV-related miRNAs and offer potential biomarkers for early prediction of liver inflammation and early-stage progression of end-stage liver disease. However, the roles of these miRNAs (miR-224-5p, miR-200a-3p) in the progression of HBV-related liver diseases remain unclear. Furthermore, the mechanisms by which these miRNAs influence the progression of HBV-related liver diseases require further in-depth investigation.

## Data Availability

The raw data supporting the conclusions of this article will be made available by the authors, without undue reservation.
