# Correction: Identification of serum MicroRNAs associated with hepatic immunoinflammatory injury in chronic hepatitis B: implications for non-invasive diagnosis

**DOI:** 10.3389/fimmu.2026.1784456

**Published:** 2026-01-16

**Authors:** Mengkui Han, Junchi Xu, Xunxun Wu, Zhiqing Hu, Xiaoyuan Hu, Xiaolong Zhu, Cailin Chen, Qinkao Xuan, Yang Li, Chuanwu Zhu, Li Zhu, Xiaohua Yang, Jin Li

**Affiliations:** 1 Department of Infectious Diseases, The Affiliated Infectious Diseases Hospital of Soochow University, Suzhou, Jiangsu, China; 2 Department of General Surgery, The Affiliated Hospital of Jiangnan University, Wuxi, Jiangsu, China; 3 Department of General Surgery, The First Affiliated Hospital of Soochow University, Suzhou, Jiangsu, China; 4 Department of Gastroenterology, Jiangsu Shengze Hospital, Suzhou, Jiangsu, China

**Keywords:** biomarkers, CHB, HBV, HCC, liver fibrosis, miRNAs

Author “Xiaohua Yang” was erroneously assigned to affiliation “Department of Gastroenterology, Jiangsu Shengze Hospital, Suzhou, Jiangsu, China”. The affiliation has been amended to the correct affiliation “Department of General Surgery, The First Affiliated Hospital of Soochow University, Suzhou, Jiangsu, China”.

Author “Xiaoyuan Hu” was erroneously assigned to affiliation “Department of General Surgery, The Affiliated Hospital of Jiangnan University, Wuxi, Jiangsu, China”. The affiliation has been amended to the correct affiliation “Department of General Surgery, The First Affiliated Hospital of Soochow University, Suzhou, Jiangsu, China”.

Author “Xiaolong Zhu” was erroneously assigned to affiliation “Department of General Surgery, The Affiliated Hospital of Jiangnan University, Wuxi, Jiangsu, China”. The affiliation has been amended to the correct affiliation “Department of General Surgery, The First Affiliated Hospital of Soochow University, Suzhou, Jiangsu, China”.

Author “Cailin Chen” was erroneously assigned to affiliation “Department of General Surgery, The First Affiliated Hospital of Soochow University, Suzhou, Jiangsu, China”. The affiliation has been amended to the correct affiliation “Department of Gastroenterology, Jiangsu Shengze Hospital, Suzhou, Jiangsu, China”.

Author “Qinkao Xuan” was erroneously assigned to affiliation “Department of Gastroenterology, Jiangsu Shengze Hospital, Suzhou, Jiangsu, China”. The affiliation has been amended to the correct affiliation “Department of General Surgery, The First Affiliated Hospital of Soochow University, Suzhou, Jiangsu, China”.

The original version of this article has been updated.

