# Supplementary material for: Identification of serum MicroRNAs associated with hepatic immunoinflammatory injury in chronic hepatitis B: implications for non-invasive diagnosis
Source: Front Immunol. 2025 Dec 11;16:1671149. doi: 10.3389/fimmu.2025.1671149 (PMC12738851; doi:10.3389/fimmu.2025.1671149)
Supplement: Supplementary file 1 [file Table1.docx]

| **Supplementary Table 1. Sequences of Primers Used for Stem-Loop RT-qPCR of miRNAs** | |
| --- | --- |
| Bulge-Loop hsa-let-7a-5p RT Primer | CTCAACTGAATTGCCGACTCCACGACACCAGTTGAGAACTAT |
| Bulge-Loop hsa-let-7a-5p Forward Primer | GGCCGTGAGGTAGTAGGTTGT |
| Bulge-Loop hsa-let-7f-5p RT Primer | CTCAACTGAATTGCCGACTCCACGACACCAGTTGAGAACTAT |
| Bulge-Loop hsa-let-7f-5p Forward Primer | GGCCGTGAGGTAGTAGATTGT |
| Bulge-Loop hsa-miR-10a-5p RT Primer | CTCAACTGAATTGCCGACTCCACGACACCAGTTGAGCACAAA |
| Bulge-Loop hsa-miR-10a-5p Forward Primer | GGCTACCCTGTAGATCCGAA |
| Bulge-Loop hsa-miR-15a-5p RT Primer | CTCAACTGAATTGCCGACTCCACGACACCAGTTGAGCTCAAA |
| Bulge-Loop hsa-miR-15a-5p Forward Primer | GCCGTAGCAGCACATAATGG |
| Bulge-Loop hsa-miR-18a-5p RT Primer | CTCAACTGAATTGCCGACTCCACGACACCAGTTGAGCTATCT |
| Bulge-Loop hsa-miR-18a-5p Forward Primer | GCCGGTAAGGTGCATCTAGT |
| Bulge-Loop hsa-miR-21-5p RT Primer | CTCAACTGAATTGCCGACTCCACGACACCAGTTGAGTCAACA |
| Bulge-Loop hsa-miR-21-5p Forward Primer | CGGCGCTAGCTTATCAGAC |
| Bulge-Loop hsa-miR-22-3p RT Primer | CTCAACTGAATTGCCGACTCCACGACACCAGTTGAGACAGTT |
| Bulge-Loop hsa-miR-22-3p Forward Primer | CGAAGCTGCCAGTTGAAGAA |
| Bulge-Loop hsa-miR-26a-5p RT Primer | CTCAACTGAATTGCCGACTCCACGACACCAGTTGAGAGCCTA |
| Bulge-Loop hsa-miR-26a-5p Forward Primer | GCGGCTTCAAGTAATCCAGG |
| Bulge-Loop hsa-miR-27a-3p RT Primer | CTCAACTGAATTGCCGACTCCACGACACCAGTTGAGGCGGAA |
| Bulge-Loop hsa-miR-27a-3p Forward Primer | CGGCTTCACAGTGGCTAAG |
| Bulge-Loop hsa-miR-29a-5p RT Primer | CTCAACTGAATTGCCGACTCCACGACACCAGTTGAGCTGAAC |
| Bulge-Loop hsa-miR-29a-5p Forward Primer | CGCGCCACTGATTTCTTTTGGT |
| Bulge-Loop hsa-miR-29c-3p RT Primer | CTCAACTGAATTGCCGACTCCACGACACCAGTTGAGTAACCG |
| Bulge-Loop hsa-miR-29c-3p Forward Primer | GCGCCTAGCACCATTTGAAAT |
| Bulge-Loop hsa-miR-30a-5p RT Primer | CTCAACTGAATTGCCGACTCCACGACACCAGTTGAGCTTCCA |
| Bulge-Loop hsa-miR-30a-5p Forward Primer | GGCCTGTAAACATCCTCGAC |
| Bulge-Loop hsa-miR-92a-3p RT Primer | CTCAACTGAATTGCCGACTCCACGACACCAGTTGAGACAGGC |
| Bulge-Loop hsa-miR-92a-3p Forward Primer | GGCTATTGCACTTGTCCCG |
| Bulge-Loop hsa-miR-99a-5p RT Primer | CTCAACTGAATTGCCGACTCCACGACACCAGTTGAGCACAAG |
| Bulge-Loop hsa-miR-99a-5p Forward Primer | CCAACCCGTAGATCCGATG |
| Bulge-Loop hsa-miR-100-5p RT Primer | CTCAACTGAATTGCCGACTCCACGACACCAGTTGAGCACAAG |
| Bulge-Loop hsa-miR-100-5p Forward Primer | GCGAACCCGTAGATCCGAA |
| Bulge-Loop hsa-miR-101-3p RT Primer | CTCAACTGAATTGCCGACTCCACGACACCAGTTGAGTTCAGT |
| Bulge-Loop hsa-miR-101-3p Forward Primer | GCCGCGTACAGTACTGTGATA |
| Bulge-Loop hsa-miR-106b-5p RT Primer | CTCAACTGAATTGCCGACTCCACGACACCAGTTGAGATCTGC |
| Bulge-Loop hsa-miR-106b-5p Forward Primer | CGGGCTAAAGTGCTGACAGT |
| Bulge-Loop hsa-miR-122-5p RT Primer | CTCAACTGAATTGCCGACTCCACGACACCAGTTGAGCAAACA |
| Bulge-Loop hsa-miR-122-5p Forward Primer | GGCTGGAGTGTGACAATGG |
| Bulge-Loop hsa-miR-125a-5p RT Primer | CTCAACTGAATTGCCGACTCCACGACACCAGTTGAGTCACAG |
| Bulge-Loop hsa-miR-125a-5p Forward Primer | CGTCCCTGAGACCCTTTAAC |
| Bulge-Loop hsa-miR-125b-5p RT Primer | CTCAACTGAATTGCCGACTCCACGACACCAGTTGAGTCACAA |
| Bulge-Loop hsa-miR-125b-5p Forward Primer | GCTCCCTGAGACCCTAAC |
| Bulge-Loop hsa-miR-126-3p RT Primer | CTCAACTGAATTGCCGACTCCACGACACCAGTTGAGCGCATT |
| Bulge-Loop hsa-miR-126-3p Forward Primer | GGCCTCGTACCGTGAGTAAT |
| Bulge-Loop hsa-miR-145-5p RT Primer | CTCAACTGAATTGCCGACTCCACGACACCAGTTGAGAGGGAT |
| Bulge-Loop hsa-miR-145-5p Forward Primer | CGGGTCCAGTTTTCCCAGGA |
| Bulge-Loop hsa-miR-146a-5p RT Primer | CTCAACTGAATTGCCGACTCCACGACACCAGTTGAGAACCCA |
| Bulge-Loop hsa-miR-146a-5p Forward Primer | CGCGGTGAGAACTGAATTCCA |
| Bulge-Loop hsa-miR-148a-3p RT Primer | CTCAACTGAATTGCCGACTCCACGACACCAGTTGAGACAAAG |
| Bulge-Loop hsa-miR-148a-3p Forward Primer | GGCTCAGTGCACTACAGAA |
| Bulge-Loop hsa-miR-155-5p RT Primer | CTCAACTGAATTGCCGACTCCACGACACCAGTTGAGAACCCC |
| Bulge-Loop hsa-miR-155-5p Forward Primer | CGGCGCTTAATGCTAATCGTGATA |
| Bulge-Loop hsa-miR-192-5p RT Primer | CTCAACTGAATTGCCGACTCCACGACACCAGTTGAGGGCTGT |
| Bulge-Loop hsa-miR-192-5p Forward Primer | GGCGGCTGACCTATGAATTG |
| Bulge-Loop hsa-miR-194-5p RT Primer | CTCAACTGAATTGCCGACTCCACGACACCAGTTGAGTCCACA |
| Bulge-Loop hsa-miR-194-5p Forward Primer | GCGTGTAACAGCAACTCCA |
| Bulge-Loop hsa-miR-199a-5p RT Primer | CTCAACTGAATTGCCGACTCCACGACACCAGTTGAGGAACAG |
| Bulge-Loop hsa-miR-199a-5p Forward Primer | GGCCCAGTGTTCAGACTAC |
| Bulge-Loop hsa-miR-200a-3p RT Primer | CTCAACTGAATTGCCGACTCCACGACACCAGTTGAGACATCG |
| Bulge-Loop hsa-miR-200a-3p Forward Primer | GCGCCTAACACTGTCTGGTAA |
| Bulge-Loop hsa-miR-221-3p RT Primer | CTCAACTGAATTGCCGACTCCACGACACCAGTTGAGGAAACC |
| Bulge-Loop hsa-miR-221-3p Forward Primer | CCGGAGCTACATTGTCTGCTG |
| Bulge-Loop hsa-miR-224-5p RT Primer | CTCAACTGAATTGCCGACTCCACGACACCAGTTGAGCTAAAC |
| Bulge-Loop hsa-miR-224-5p Forward Primer | GCGCGGCTCAAGTCACTA |
| Bulge-Loop hsa-miR-638 RT Primer | CTCAACTGAATTGCCGACTCCACGACACCAGTTGAGAGGCCG |
| Bulge-Loop hsa-miR-638 Forward Primer | CAGGGATCGCGGGCGGGTGG |
| Bulge-Loop hsa-miR-885-5p RT Primer | CTCAACTGAATTGCCGACTCCACGACACCAGTTGAGAGAGGC |
| Bulge-Loop hsa-miR-885-5p Forward Primer | GGCGTCCATTACACTACCCT |
| Bulge-Loop hsa-miR-939-5p RT Primer | CTCAACTGAATTGCCGACTCCACGACACCAGTTGAGCACCCC |
| Bulge-Loop hsa-miR-939-5p Forward Primer | GGTGGGGAGCTGAGGCTCT |
| **The reverse primer (Bulge-Loop miR-Reverse Primer) is universal and used for all miRNA amplifications** | |
| Bulge-Loop miR-Reverse Primer | CAACTGGTGTCGTGGAGTCG |
| Note: This table lists the nucleotide sequences (5′→3′) of primers employed in the stem-loop reverse transcription quantitative polymerase chain reaction (RT-qPCR) assay for quantification of mature miRNA expression levels. For each target miRNA, three primer types are provided:  RT primer: miRNA-specific reverse transcription primer with a bulge-loop structure to enhance specificity and efficiency.  Forward primer: miRNA-specific forward amplification primer.  Reverse primer: The reverse primer, listed at the end of the table, is a universal primer used across all miRNA assays.  All primers were designed using the Bulge-Loop™ miRNA qPCR Primer Set (All primers were designed using the Bulge-Loop™ miRNA qPCR Primer Set (RiboBio Co., Ltd., Guangzhou, China; GeneAdv Co., Ltd., Suzhou, China). The reverse transcription step was performed using stem-loop RT primers, followed by SYBR Green-based real-time PCR with miRNA-specific forward and universal reverse primers. | |

| **Supplementary Table 2 The comparison of serum miRNAs between HBV infection group and healthy controls.** | | | | |
| --- | --- | --- | --- | --- |
| **miRNAs** | **Healthy controls (n=42)** | **HBV infection group (n=123)** | **Fold change*** | **P value#** |
| miR-224-5p | 0.72 (0.55-0.93) | 3.38 (1.84-25.45) | 16.012 | 1.10E-20 |
| miR-99a-5p | 0.16 (0.09-0.22) | 0.45 (0.20-0.94) | 10.337 | 0.043 |
| miR-15a-5p | 3.85 (1.78 – 8.02) | 4.80 (1.89 – 24.78) | 6.122 | 0.009 |
| miR-26a-5p | 1.07 (0.56-2.17) | 2.33 (0.98-5.62) | 5.233 | 0.0648 |
| miR-125a-5p | 1.13 (0.87-1.59) | 1.50 (1.02-11.88) | 4.949 | 7.84E-05 |
| miR-122-5p | 21.48 (15.37-32.06) | 33.76 (16.91-74.83) | 4.642 | 0.0626 |
| miR-27a-3p | 0.90 (0.58-1.72) | 1.33 (0.82-3.89) | 3.447 | 0.1407 |
| miR-18a-5p | 65.43 (55.51 – 75.42) | 179.60 (150.80-220.30) | 3.382 | 1.15E-18 |
| miR-30a-5p | 2.88 (2.38-5.55) | 4.51 (2.50-17.57) | 3.288 | 0.0006 |
| miR-101-3p | 0.77 (0.45-1.71) | 2.00 (1.48-4.19) | 3.260 | 0.0334 |
| miR-200a-3p | 2.63 (2.35-3.39) | 2.72 (2.05-11.67) | 2.735 | 0.0015 |
| miR-22-3p | 1.23 (0.84-2.24) | 1.62 (0.43-4.19) | 2.365 | 0.019 |
| miR-29a-5p | 4.01 (2.75-5.37) | 7.29 (3.03-12.50) | 2.154 | 5.72E-05 |
| miR-194-5p | 1.17 (0.92-1.54) | 0.73 (0.35-2.24) | 2.136 | 0.2129 |
| miR-192-5p | 3.89 (2.90-4.46) | 3.63 (2.20-7.27) | 2.078 | 0.0681 |
| miR-125b-5p | 3.78 (2.75-4.80) | 4.40 (3.26-6.81) | 2.033 | 0.0671 |
| miR-106b-5p | 3.42 (2.03-6.32) | 6.90 (3.66-9.64) | 2.029 | 0.014 |
| miR-939-5p | 0.07 (0.06-0.09) | 0.11 (0.08-0.16) | 1.883 | 9.85E-08 |
| Let-7f-5p | 8.18 (6.18 – 10.77) | 13.76 (9.74 – 18.12) | 1.695 | 2.89E-08 |
| miR-148a-3p | 1.85 (1.26-2.36) | 1.71 (0.93-3.29) | 1.539 | 0.1569 |
| miR-10a-5p | 16.21 (13.45 – 19.07) | 23.88 (18.32 – 28.05) | 1.483 | 5.64E-11 |
| miR-92a-3p | 102.8 (54.60-191.70) | 89.02 (54.21-152.90) | 1.112 | 0.6786 |
| miR-21-5p | 27.43 (17.66-47.99) | 29.49 (15.43-49.98) | 1.079 | 0.5664 |
| miR-100-5p | 11.09 (8.97-12.99) | 10.81 (9.73-13.38) | 1.057 | 0.3979 |
| miR-146a-5p | 15.35 (9.66-18.06) | 15.25 (12.17-19.25) | 1.057 | 0.4128 |
| miR-221-3p | 7.30 (5.27-9.37) | 6.50 (4.28-9.00) | 0.972 | 0.7548 |
| Let-7a-5p | 85.4 (68.2 – 110.6) | 82.2 (55.4 – 112.1) | 0.919 | 0.2626 |
| miR-155-5p | 1.31 (1.10-1.91) | 1.11 (0.80-1.37) | 0.762 | 2.89E-05 |
| miR-638 | 772.3 (506.1-1083) | 493.5 (159.2-889.1) | 0.739 | 0.0071 |
| miR-29c-3p | 9.06 (5.33-13.13) | 4.18 (2.00-7.24) | 0.714 | 0.1022 |
| miR-199a-5p | 1.49 (1.08-1.78) | 0.97 (0.81-1.15) | 0.69 | 8.37E-09 |
| miR-126-3p | 36.35 (25.98-47.08) | 20.40 (15.29-27.51) | 0.59 | 6.17E-11 |
| miR-885-5p | 51.41 (38.68-66.28) | 27.82 (18.29-38.61) | 0.587 | 1.21E-11 |
| miR-145-5p | 20.97 (16.73-34.45) | 4.57 (2.97-8.71) | 0.265 | 3.59E-14  - |
| Note: Data were median (25% percentile – 75% percentile). Abbreviations: HBV infection group, include Chronic hepatitis B、HBV-Cirrhosis、HBV-Hepatocellular carcinoma. *: Fold change between two groups was calculated based on the mean value of each miRNA. The normality analysis was conducted using D'Agostino & Pearson omnibus normality test; #: non-normal Gaussian distribution, Mann Whitney test was used. Statistical significance was defined as p < 0.05. P-values are reported in scientific notation (e.g., 4.34E-04 = 0.000434). | | | | |

| **Supplementary Table 3 Global miRNA Expression Profiles in the Cohort of HBV-Associated Advanced Liver Disease** | | | | | |
| --- | --- | --- | --- | --- | --- |
| **miRNAs** | **Healthy controls**  **(n=42)** | **CHB**  **(n=40)** | **Cirrhosis**  **(n=42)** | **HCC**  **(n=41)** | **P value** |
| miR-224-5p | 0.72(0.55-0.93) | 1.77(1.47-1.92) | 29.46(19.78-35.13) | 4.08(2.88-5.76) | 6.39E-27 |
| miR-18a-5p | 65.43(55.51-75.42) | 175.30(154.20-194.80) | 189.70(97.09-379.40) | 185.70(153.60-214.50) | 8.21E-17 |
| miR-125a-5p | 1.13(0.87-1.60) | 1.16(0.81-1.34) | 13.85(9.36-18.70) | 1.23(0.92-1.95) | 4.95E-16 |
| miR-145-5p | 20.97(16.73-34.45) | 3.66(2.45-4.74) | 4.54(3.15-8.79) | 7.46(4.77-11.40) | 3.15E-15 |
| miR-27a-3p | 0.90(0.58-1.72) | 0.88(0.49-1.08) | 1.35(0.81-2.52) | 5.66(2.82-10.96) | 3.69E-12 |
| miR-939-5p | 0.07(0.06-0.09) | 0.09(0.08-0.11) | 0.10(0.07-0.12) | 0.22(0.14-0.28) | 4.08E-12 |
| miR-126-3p | 36.35(25.98-47.08) | 16.37(11.64-21.17) | 21.27(16.70-27.38) | 27.00(17.88-35.81) | 1.08E-11 |
| miR-30a-5p | 2.88(2.38-5.55) | 3.05(2.48-4.73) | 19.76(10.78-35.60) | 3.46(1.87-8.25) | 4.77E-11 |
| miR-22-3p | 1.22(0.84-2.24) | 0.59(0.34-1.25) | 4.61(2.07-10.78) | 1.29(0.39-2.61) | 5.14E-11 |
| miR-15a-5p | 3.85(1.77-8.01) | 2.15(0.98-4.44) | 35.24(11.15-135.80) | 4.39(2.20-15.85) | 9.05E-11 |
| miR-200a-3p | 2.63(2.35-3.30) | 2.28(1.77-2.67) | 2.50(1.85-3.05) | 19.24(10.18-24.92) | 1.78E-10 |
| miR-26a-5p | 1.07(0.55-2.17) | 1.10(0.79-2.38) | 2.05(0.81-3.23) | 6.25(2.28-15.38) | 3.35E-10 |
| miR-106b-5p | 3.42(2.03-6.32) | 8.11(6.99-12.58) | 6.89(4.02-9.06) | 3.41(2.30-6.76) | 4.9E-10 |
| miR-885-5p | 51.41(38.68-66.28) | 24.57(18.40-33.67) | 33.57(19.04-41.35) | 26.19(17.13-37.98) | 1.02E-09 |
| miR-99a-5p | 0.16(0.10-0.22) | 0.74(0.29-1.01) | 0.47(0.16-0.86) | 0.34(0.18-0.93) | 3.06E-09 |
| miR-10a-5p | 16.21(13.45-19.07) | 26.46(19.58-30.63) | 23.26(17.94-28.50) | 23.51(17.85-26.30) | 4.67E-09 |
| let-7f-5p | 8.18(6.18-10.77) | 15.75(12.82-19.84) | 13.60(9.73-19.40) | 11.00(7.71-16.07) | 8.91E-09 |
| miR-101-3p | 0.77(0.45-1.71) | 2.05(1.70-4.11) | 2.12(1.44-4.14) | 1.88(1.18-4.68) | 8.23E-08 |
| miR-199a-5p | 1.49(1.08-1.68) | 0.92(0.77-1.04) | 0.99(0.81-1.26) | 1.02(0.82-1.17) | 2.89E-07 |
| miR-221-3p | 7.30(5.27-9.37) | 4.42(3.47-6.39) | 8.25(5.47-10.71) | 7.22(5.82-10.79) | 2.28E-06 |
| miR-146a-5p | 15.35(9.66-18.06) | 12.6(9.0-15.4) | 18.1(14.8-21.0) | 15.6(12.4-19.3) | 4.05E-04 |
| miR-29a-5p | 4.01(2.75-5.37) | 5.87(3.94-9.63) | 11.88(3.05-15.70) | 4.98(2.33-12.67) | 0.0002 |
| miR-29c-3p | 9.06(5.33-13.13) | 3.55(1.86-6.94) | 4.99(1.94-7.42) | 4.06(2.19-7.66) | 0.0003 |
| miR-148a-3p | 1.85(1.26-2.36) | 2.24(1.61-3.87) | 1.94(0.94-3.16) | 1.14(0.51-2.31) | 0.0007 |
| miR-122-5p | 21.48(15.37-32.06) | 46.81(22.19-85.17) | 35.14(19.13-78.82) | 19.31(10.19-72.84) | 0.0009 |
| miR-100-5p | 11.09(8.97-12.99) | 11.06(9.81-13.32) | 11.23(10.44-14.20) | 9.81(8.78-11.32) | 0.0023 |
| miR-155-5p | 1.31(1.10-1.91) | 1.05(0.73-1.33) | 1.18(0.90-1.41) | 1.11(0.72-1.39) | 0.0041 |
| miR-638 | 772.30(506.10-1083.00) | 506.70(272.40-854.80) | 582.80(193.00-1088.00) | 301.80(132.90-762.10) | 0.0053 |
| let-7a-5p | 85.40(68.24-110.60) | 68.76(40.67-96.84) | 90.96(67.87-122.20) | 73.02(50.48-108.80) | 0.0136 |
| miR-125b-5p | 3.78(2.75-4.80) | 4.29(3.26-7.57) | 4.75(3.45-6.76) | 4.04(2.63-6.99) | 0.0981 |
| miR-194-5p | 1.17(0.92-1.54) | 0.98(0.32-2.23) | 0.84(0.40-2.12) | 0.59(0.37-2.31) | 0.1661 |
| miR-192-5p | 3.89(2.90-4.45) | 3.1(2.08-6.92) | 4.67(2.73-7.35) | 3.03(1.71-8.74) | 0.2204 |
| miR-92a-3p | 102.80(54.60-191.70) | 71.94(54.41-126.70) | 102.80(53.93-176.80) | 94.41(53.88-185.70) | 0.6052 |
| miR-21-5p | 27.43(17.66-47.99) | 30.23(17.69-41.99) | 31.39(17.44-55.02) | 26.66(13.92-53.36) | 0.6138 |
| Note: Data were median (25% percentile – 75% percentile). Abbreviations: HBV infection group, include Chronic hepatitis B、HBV-Cirrhosis、HBV-Hepatocellular carcinoma. The Kruskal-Wallis test was used for the comparison among the four groups. Statistical significance was defined as p < 0.05. P-values are reported in scientific notation (e.g., 4.34E-04 = 0.000434). | | | | | |

| **Supplementary Table 4. Multiple comparisons of inter-group miRNA expression levels and results after correction** | | | | | | | | | | | | |
| --- | --- | --- | --- | --- | --- | --- | --- | --- | --- | --- | --- | --- |
| **miRNA** | **HC vs CHB** | | **HC vs Cirrhosis** | | **HC vs HCC** | | **CHB vs Cirrhosis** | | **CHB vs HCC** | | **Cirrhosis vs HCC** | |
|  | **p-value** | **FDR** | **p-value** | **FDR** | **p-value** | **FDR** | **p-value** | **FDR** | **p-value** | **FDR** | **p-value** | **FDR** |
| let-7a-5p | 0.023 | ns | ns | ns | ns | ns | 0.003 | 0.016 | ns | ns | ns | ns |
| let-7f-5p | <0.001 | <0.001 | <0.001 | <0.001 | 0.016 | ns | ns | ns | <0.001 | 0.004 | 0.034 | ns |
| miR-10a-5p | <0.001 | <0.001 | <0.001 | <0.001 | <0.001 | <0.001 | ns | ns | 0.025 | ns | ns | ns |
| miR-15a-5p | 0.031 | ns | <0.001 | <0.001 | ns | ns | <0.001 | <0.001 | 0.003 | 0.039 | <0.001 | <0.001 |
| miR-18a-5p | <0.001 | <0.001 | <0.001 | <0.001 | <0.001 | <0.001 | ns | ns | ns | ns | ns | ns |
| miR-21-5p | ns | ns | ns | ns | ns | ns | ns | ns | ns | ns | ns | ns |
| miR-22-3p | <0.001 | ns | <0.001 | <0.001 | ns | ns | <0.001 | <0.001 | ns | ns | <0.001 | <0.001 |
| miR-26a-5p | ns | ns | 0.017 | ns | <0.001 | <0.001 | ns | ns | <0.001 | <0.001 | <0.001 | <0.001 |
| miR-27a-3p | ns | ns | ns | ns | <0.001 | <0.001 | <0.001 | 0.028 | <0.001 | <0.001 | <0.001 | <0.001 |
| miR-29a-5p | <0.001 | ns | <0.001 | <0.001 | ns | ns | 0.008 | ns | ns | ns | ns | ns |
| miR-29c-3p | <0.001 | <0.001 | <0.001 | 0.011 | <0.001 | 0.003 | ns | ns | ns | ns | ns | ns |
| miR-30a-5p | ns | ns | <0.001 | <0.001 | ns | ns | <0.001 | <0.001 | ns | ns | <0.001 | <0.001 |
| miR-92a-3p | ns | ns | ns | ns | ns | ns | ns | ns | ns | ns | ns | ns |
| miR-99a-5p | <0.001 | <0.001 | <0.001 | <0.001 | <0.001 | <0.001 | 0.049 | ns | 0.016 | ns | ns | ns |
| miR-100-5p | ns | ns | ns | ns | ns | ns | ns | ns | 0.01 | ns | <0.001 | 0.001 |
| miR-101-3p | <0.001 | <0.001 | <0.001 | <0.001 | <0.001 | <0.001 | ns | ns | ns | ns | ns | ns |
| miR-106b-5p | <0.001 | <0.001 | <0.001 | 0.002 | ns | ns | 0.004 | ns | <0.001 | <0.001 | 0.002 | 0.019 |
| miR-122-5p | <0.001 | 0.002 | 0.005 | ns | ns | ns | ns | ns | 0.011 | 0.021 | ns | ns |
| miR-125a-5p | ns | ns | <0.001 | <0.001 | ns | ns | <0.001 | <0.001 | ns | ns | <0.001 | <0.001 |
| miR-125b-5p | 0.039 | ns | 0.005 | ns | ns | ns | ns | ns | ns | ns | ns | ns |
| miR-126-3p | <0.001 | <0.001 | <0.001 | <0.001 | 0.002 | 0.013 | 0.003 | ns | <0.001 | <0.001 | 0.028 | ns |
| miR-145-5p | <0.001 | <0.001 | <0.001 | <0.001 | <0.001 | <0.001 | 0.011 | ns | <0.001 | 0.002 | 0.025 | ns |
| miR-146a-5p | 0.045 | ns | ns | ns | ns | ns | <0.001 | <0.001 | 0.002 | 0.015 | ns | ns |
| miR-148a-3p | 0.022 | ns | ns | ns | 0.008 | ns | ns | ns | <0.001 | <0.001 | 0.019 | ns |
| miR-155-5p | 0.002 | 0.009 | 0.031 | ns | 0.002 | 0.011 | ns | ns | ns | ns | ns | ns |
| miR-192-5p | ns | ns | ns | ns | ns | ns | ns | ns | ns | ns | ns | ns |
| miR-194-5p | ns | ns | ns | ns | 0.034 | ns | ns | ns | ns | ns | ns | ns |
| miR-199a-5p | <0.001 | <0.001 | <0.001 | 0.001 | <0.001 | <0.001 | ns | ns | ns | ns | ns | ns |
| miR-200a-3p | 0.003 | ns | ns | ns | <0.001 | <0.001 | ns | ns | <0.001 | <0.001 | <0.001 | <0.001 |
| miR-221-3p | <0.001 | <0.001 | ns | ns | ns | ns | <0.001 | <0.001 | <0.001 | <0.001 | ns | ns |
| miR-224-5p | <0.001 | <0.001 | <0.001 | <0.001 | <0.001 | <0.001 | <0.001 | <0.001 | <0.001 | <0.001 | <0.001 | ns |
| miR-638 | 0.02 | ns | ns | ns | <0.001 | 0.003 | ns | ns | ns | ns | 0.049 | ns |
| miR-885-5p | <0.001 | <0.001 | <0.001 | <0.001 | <0.001 | <0.001 | 0.028 | ns | ns | ns | ns | ns |
| miR-939-5p | 0.002 | ns | <0.001 | 0.019 | <0.001 | <0.001 | ns | ns | <0.001 | <0.001 | <0.001 | <0.001 |
| Note: The table summarizes pairwise comparisons of serum miRNA expression levels across five clinical groups: HC, CHB, Cirrhosis, and HCC. P-values were calculated using nonparametric tests (Kruskal-Wallis with Dunn’s post hoc) and adjusted for multiple comparisons using the False Discovery Rate (FDR) method (Benjamini-Hochberg procedure). Abbreviations: ns: not significant (p > 0.05 after FDR correction). FDR: false discovery rate-adjusted p-value. HC: healthy controls; CHB: chronic hepatitis B; HCC: hepatocellular carcinoma. | | | | | | | | | | | | |

| **Supplementary Table 5 Univariate and Multivariate Stepwise Logistic Regression Analyses for CHB versus Cirrhosis** | | | | | | |
| --- | --- | --- | --- | --- | --- | --- |
| **Variable** | **Univariate Analysis** | | | **Multivariate Analysis** | | |
|  | **OR** | **(95%CI)** | **P value** | **OR** | **(95%CI)** | **P value** |
| miR-15a-5p | 1.137 | 1.086-12.577 | 4.34E-04 |  |  |  |
| miR-22-3p | 2.367 | 0.053-3.888 | 6.50E-05 |  |  |  |
| miR-30a-5p | 1.19 | 1.106-1.309 | 2.80E-05 |  |  |  |
| miR-125a-5p | 3.573 | 1.982-29.416 | 1.30E-04 |  |  |  |
| miR-224-5p | 1.297 | 1.16-1087.38 | 2.00E-03 | 21.83 | 2.165-220.163 | 9.00E-03 |
| Note: Univariate and multivariate stepwise logistic regression analyses identifying predictors of cirrhosis in CHB patients. odds ratio. 95% CI: 95% confidence interval. Statistical significance was defined as p < 0.05. P-values are reported in scientific notation (e.g., 4.34E-04 = 0.000434). | | | | | | |

| **Supplementary Table 6 Expression Levels of Differentially Expressed miRNAs and Diagnostic Performance of Individual Biomarkers for Differentiating CHB from Cirrhosis in the discovery cohort** | | | | | | | |
| --- | --- | --- | --- | --- | --- | --- | --- |
| **Variable** | **Cutoff** | **SEN** | **SPE** | **PPV** | **NPV** | **AUC** |  |
| miR-224-5p | 2.93 | 85.40% | 100% | 100% | 87.0% | 0.973 |  |
| FIB-4 | 2.393 | 63.4% | 85.0% | 81.2% | 69.4% | 0.809 |  |
| APRI | 0.49 | 85.4% | 67.5% | 72.9% | 81.8% | 0.803 |  |
| RDW-SD | 43.2 | 73.2% | 70.0% | 71.4% | 71.8% | 0.781 |  |
| RDW-CV | 12.85 | 68.3% | 67.5% | 68.3% | 67.5% | 0.738 |  |
| Note: OR: odds ratio. 95% CI: 95% confidence interval. APRI: AST-to-platelet ratio index. FIB-4: fibrosis-4 index. RDW-SD: red cell distribution width – standard deviation. RDW-CV: red cell distribution width – coefficient of variation. SEN: sensitivity; SPE: specificity; PPV: positive predictive value; NPV: negative predictive value. Statistical significance was defined as p < 0.05. P-values are reported in scientific notation (e.g., 4.34E-04 = 0.000434). | | | | | | | |

| **Supplementary Table 7 Basic Characteristics of Patients in the External Validation Cohort** | | | | | |
| --- | --- | --- | --- | --- | --- |
| **Variables** | **HC (n=40)** | **CHB (n=35)** | **Cirrhosis (n=33)** | **HCC (n=35)** | **P Value** |
| Age (year) | 46.0(40.3-56.3) | 35.0(27.0-38.0) | 39.0(32.5-47) | 60.0(54.0-65.0) | 1.2E-14^a^ |
| Gender (Male/Female) | 27/13 | 19/16 | 29/4 | 23/12 | 0.0271^b^ |
| HBsAg (log_10_IU/ml) | - | 3.9(3.4-4.2) | 3.5(3.0-3.7) | 2.4(1.9-3.1) | 1.2E-08^a^ |
| HBV DNA (log_10_copies/ml) | - | 6.6(6.2-7.5) | 5.2(3.9-6.2) | 3.3(2.7-3.8) | 1.9E-07^a^ |
| ALT (U/L) | 19.0(13.0-27.0) | 138.0(59.0-332.0) | 46.5(29.3-115.5) | 37.3(21.8-55.7) | 4.3E-04^c^ |
| AST (U/L) | 18.5(15.0-27.8) | 77.0(46.0-218.0) | 36.0(25.0-88.0) | 32.0(24.0-54.1) | 1.8E-02^c^ |
| G **(**0/1/2/3/4) | - | 0/1/31/0/0 | 0/2/20/11/0 | 0/4/10/17/4 | 2.6E-08^b^ |
| S (0/1/2/3/4) | - | 0/6/20/9/0 | 0/0/0/0/33 | 0/2/9/7/17 | 7.1E-16^b^ |
| AFP (μg/L) | 2.7(2.2-3.1) | 5.8(4.1-12.3) | 7.8(4.5-48.2) | 11.9(3.6-1194.0) | 0.2932^c^ |
| TBil (μmol/L) | 13.2(9.7-14.8) | 11.9(9.2-18.2) | 18.0(11.9-25.8) | 20.4(14.3-29.4) | 1.1E-02^c^ |
| AlB (g/L) | 46.2(44.0-47.9) | 41.9(39.2-44.8) | 38.9(36.2-41.3) | 39.5(35.9-41.6) | 1.6E-03^c^ |
| ALP (U/L) | 72.5(63.3-78.0) | 91.0(80.0-111.0) | 86.0(69.5-116.5) | 83.6(71.8-94.8) | 0.2176^c^ |
| Note: Data are median (25% percentile – 75% percentile) except gender and G\S stage. Abbreviations: HC, Healthy control; CHB, chronic hepatitis B; Cirrhosis, HBV with cirrhosis patients; HCC, HBV with hepatocellular carcinoma patients; HBsAg, surface antigen of hepatitis B virus; HBV DNA, hepatitis B virus deoxyribonucleic acid; TBil, total bilirubin; ALT, alanine aminotransferase; AST, aspartate aminotransferase; AFP, alpha fetoprotein; ALB, albumin; ALP, alkaline phosphatase; G, inflammation stage; S, fibrosis stage. a: Kruskal-Walli’s test; b: Chi-square test; c, an ordinary one-way ANOVA test was performed, with the comparison limited to the CHB, Cirrhosis, and HCC groups. | | | | | |

| **Supplementary Table 8 Univariate and Multivariate Stepwise Logistic Regression Analyses for Predicting HCC in Patients with Chronic Hepatitis B or Cirrhosis** | | | | | | |
| --- | --- | --- | --- | --- | --- | --- |
| **Variable** | **Univariate Analysis** | | | **Multivariate Analysis** | | |
|  | **OR** | **(95%CI)** | **P value** | **OR** | **(95%CI)** | **P value** |
| miR-26a-5p | 1.233 | 1.099-1.384 | 3.68E-04 |  |  |  |
| miR-27a-3p | 1.975 | 1.509-2.586 | 7.23E-07 |  |  |  |
| miR-200a-3p | 1.559 | 1.223-1.988 | 3.36E-04 | 1.559 | 1.233-1.988 | 3.36E-04 |
| miR-939-5p | 7.99E+09 | 1.33E+06-4.81E+13 | 2.82E-07 |  |  |  |
| Note: This table presents univariate and multivariate stepwise logistic regression analyses to identify predictors of HCC among patients with chronic hepatitis B or liver cirrhosis. In the univariate analysis, all candidate miRNAs were assessed for association with HCC. Variables with p < 0.10 were entered into the multivariate stepwise model (forward selection, entry p = 0.05, removal p = 0.10). Abbreviations: OR: odds ratio; 95% CI: 95% confidence interval; CHB: chronic hepatitis B; HCC: hepatocellular carcinoma. Statistical significance was defined as p < 0.05. P-values are reported in scientific notation (e.g., 3.68E-04 = 0.000368). | | | | | | |

| **Supplementary Table 9 Diagnostic accuracy of miR-200a-3p, AFP, and combined model for HCC prediction in CHB/Cirrhosis cohort** | | | | | | |
| --- | --- | --- | --- | --- | --- | --- |
| **Variable** | **Cutoff** | **SEN** | **SPE** | **PPV** | **NPV** | **AUC** |
| Combine model | 0.341 | 0.976 | 1 | 1 | 0.988 | 0.977 |
| miR-200a-3p | 4.583 | 0.805 | 1 | 1 | 0.911 | 0.853 |
| AFP | 19.934 | 0.634 | 0.915 | 0.788 | 0.833 | 0.737 |
| Note: AFP: alpha-fetoprotein; Combine model: miR-200a-3p+AFP; SEN: sensitivity; SPE: specificity; PPV: positive predictive value; NPV: negative predictive value. | | | | | | |

**
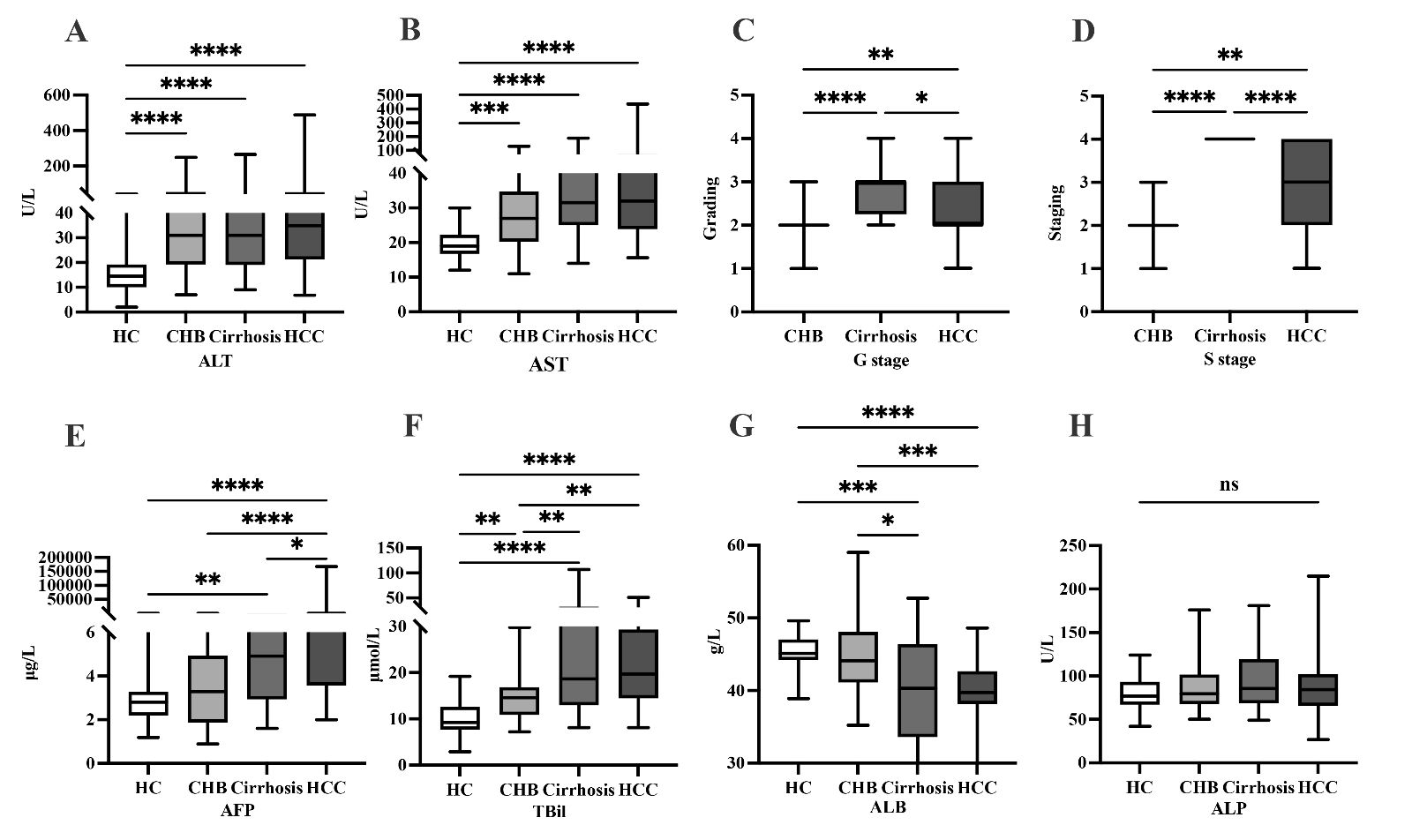
**

**Supplementary Figure 1.** Comparison of Serum Biochemical Parameters and Histopathological Staging across HC, CHB, Cirrhosis, and HCC Groups. Abbreviations: HC, Healthy control; CHB, chronic hepatitis B; Cirrhosis, HBV with cirrhosis patients; HCC, HBV with hepatocellular carcinoma patient.TBil, total bilirubin; ALT, alanine aminotransferase; AST, aspartate aminotransferase; AFP, alpha fetoprotein; ALB, albumin; ALP, alkaline phosphatase; G, inflammation stage; S, fibrosis stage. **** p < 0.0001, *** p < 0.001, ** p < 0.01, * p < 0.05.


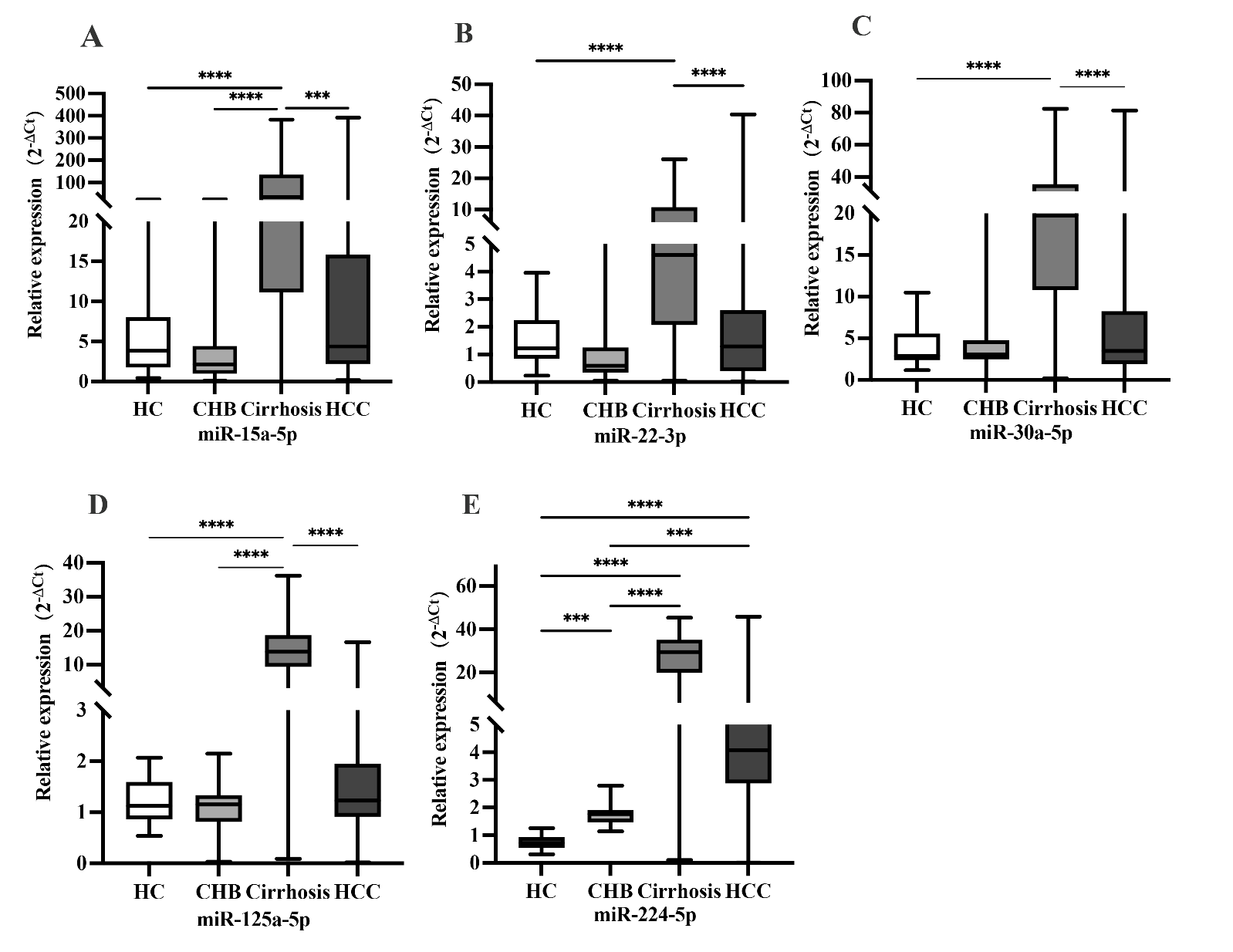

**Supplementary Figure 2.** Relative Expression Levels of Five Differentially Expressed miRNAs across HC, CHB, Cirrhosis, and HCC Groups. Statistical significance was assessed using nonparametric tests (Kruskal-Wallis test) and adjusted for multiple comparisons. Abbreviations: HC, Healthy control; CHB, chronic hepatitis B; Cirrhosis, HBV with cirrhosis patients; HCC, HBV with hepatocellular carcinoma patients. *** p < 0.001; **** p < 0.0001.


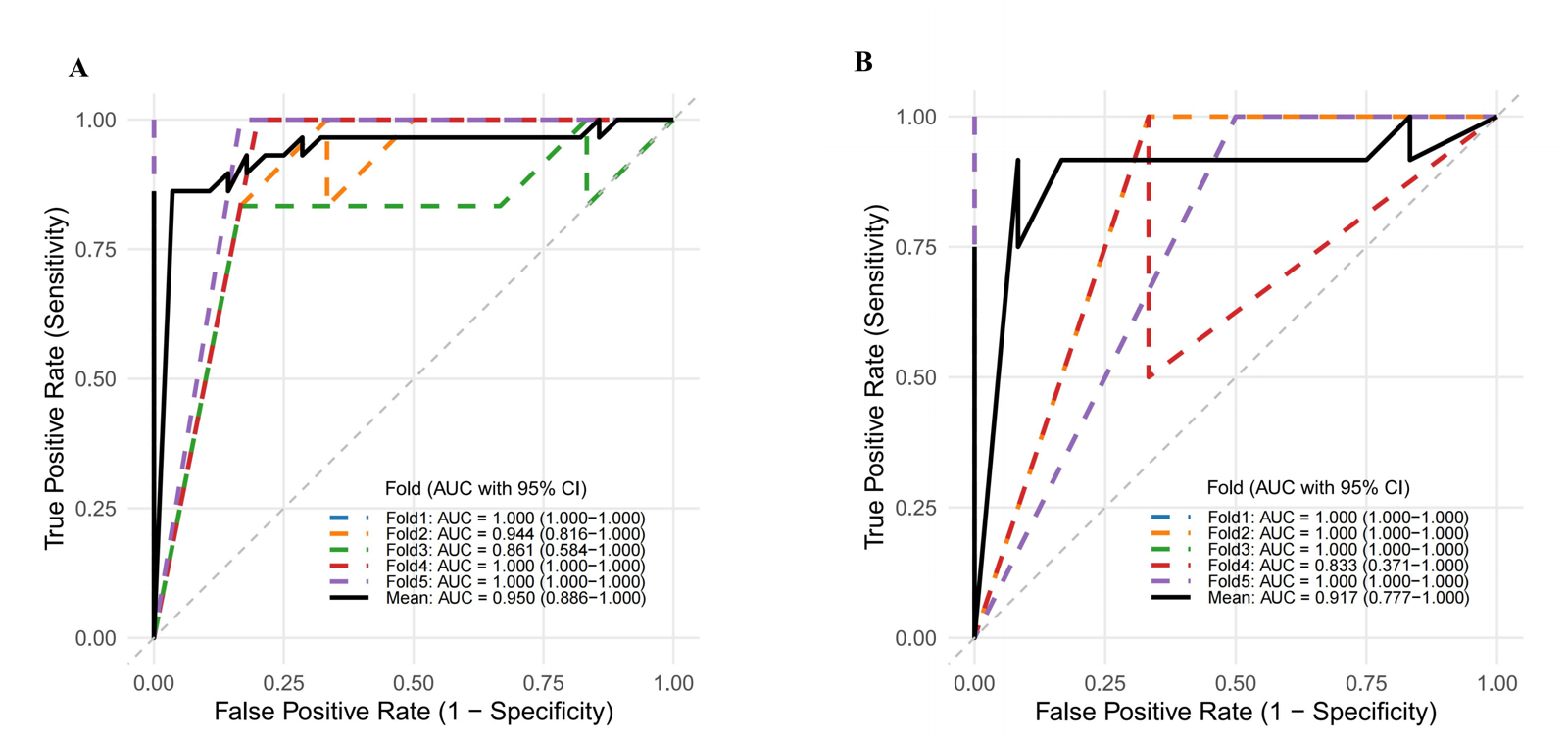


**Supplementary Figure 3.** Receiver Operating Characteristic (ROC) Curves of the Diagnostic Model for Cirrhosis Prediction in CHB Patients Using 5-Fold Cross-Validation：(A) ROC curves generated from 5-fold internal cross-validation on the training set, with individual fold performance (Fold1–Fold5) and mean AUC (95% CI: 0.950, 0.886–1.000). (B) ROC curves from an independent validation set (30% hold-out), showing model generalizability with mean AUC = 0.917 (95% CI: 0.777–1.000). The black solid line represents the mean ROC curve across all folds or the final model performance; colored dashed lines represent individual fold results. AUC values close to 1.0 indicate excellent diagnostic accuracy. All analyses were performed using R software with the pROC package.

**
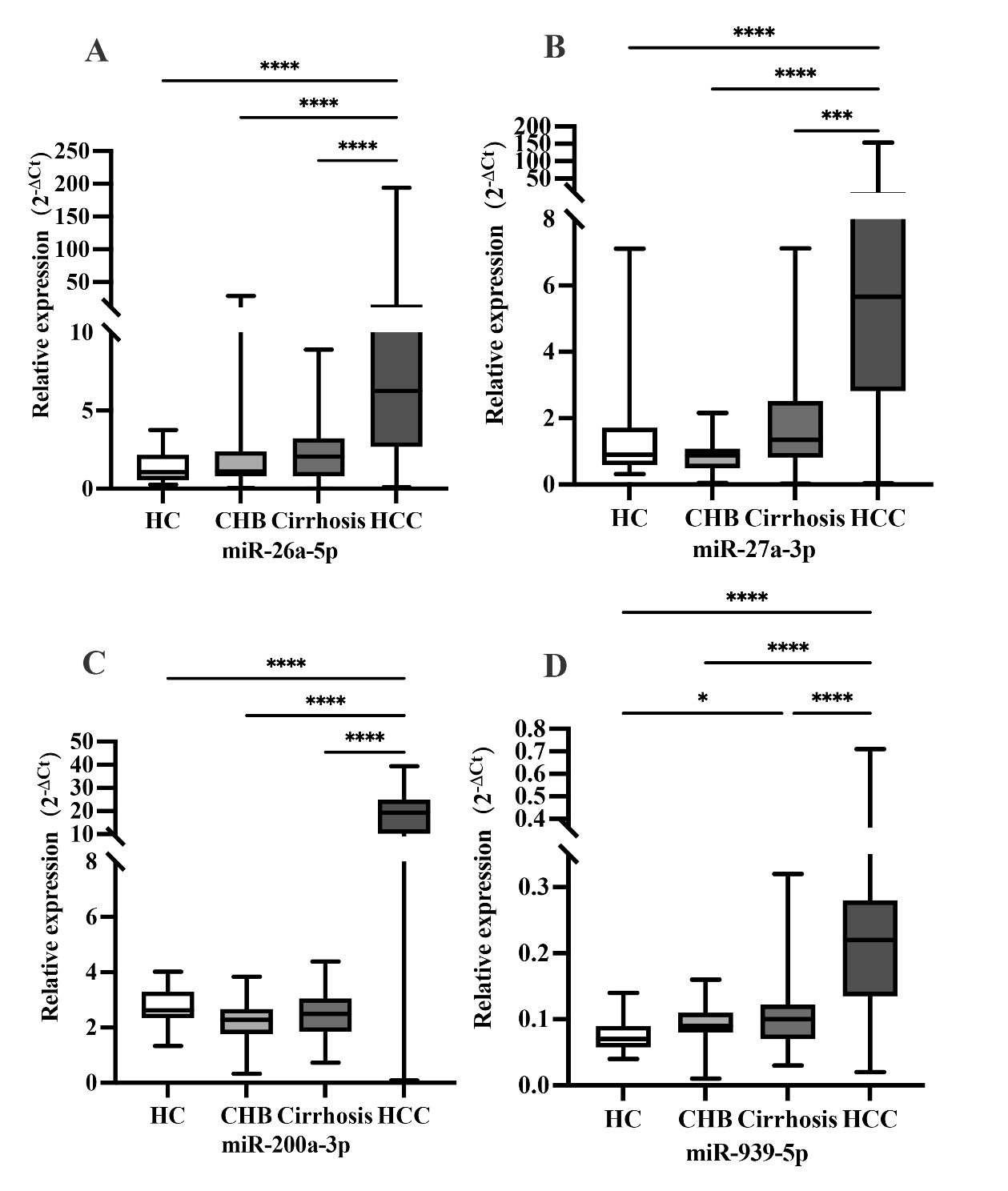
**
**Supplementary Figure 4.** Relative Expression Levels of Four Differentially Expressed miRNAs across HC, CHB, Cirrhosis, and HCC Groups. Statistical significance was assessed using nonparametric tests (Kruskal-Wallis test) and adjusted for multiple comparisons. All four miRNAs exhibited significant dysregulation across disease stages. Abbreviations: HC, Healthy control; CHB, chronic hepatitis B; Cirrhosis, HBV with cirrhosis patients; HCC, HBV with hepatocellular carcinoma patients. * p < 0.05; **** p < 0.0001.

**
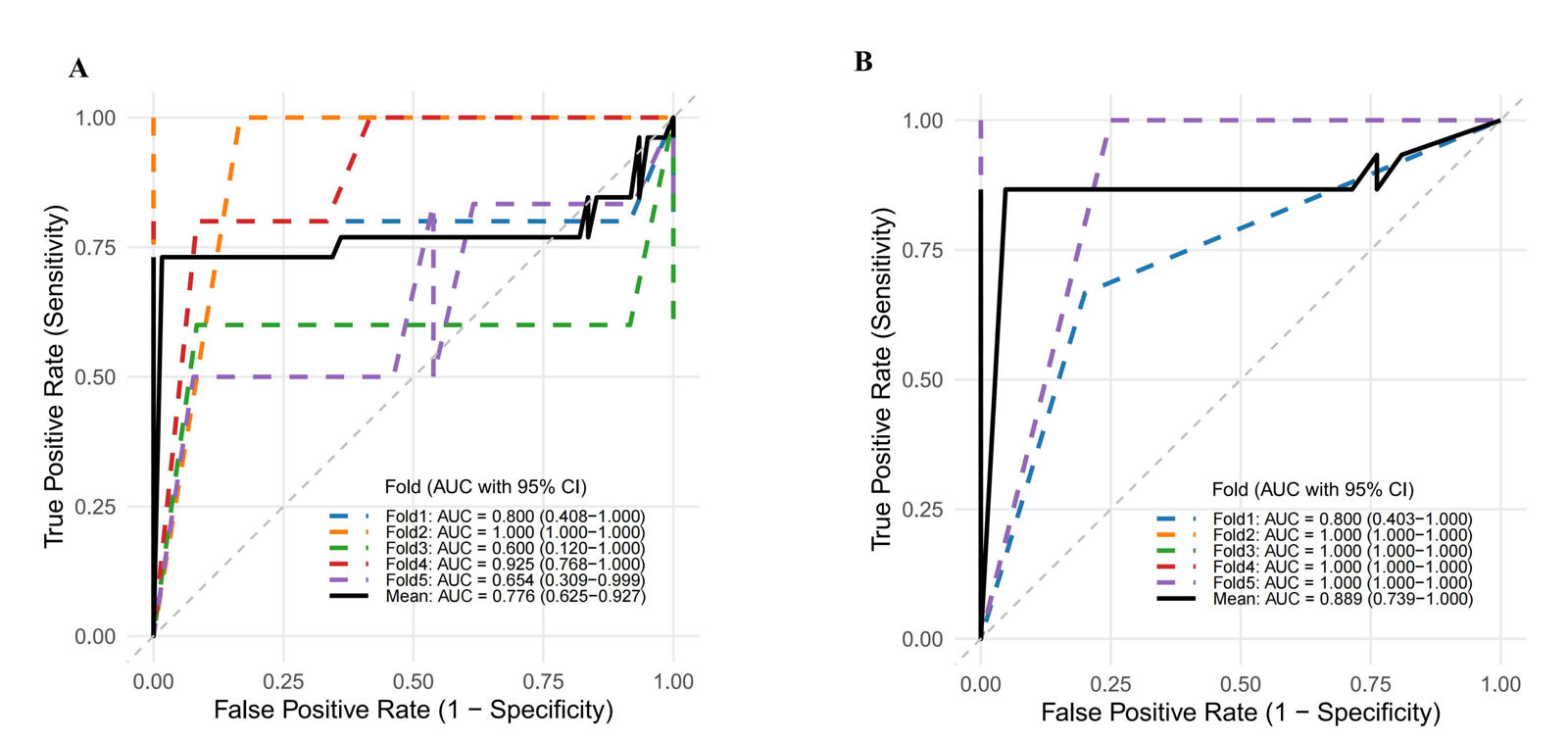
**

**Supplementary Figure 5.** Supplementary Figure 5. Receiver Operating Characteristic (ROC) Curves of the Diagnostic Model for Predicting Hepatocellular Carcinoma (HCC) in Patients with Chronic Hepatitis B or Cirrhosis Using 5-Fold Cross-Validation：(A) ROC curves generated from 5-fold internal cross-validation on the training set, showing individual fold performance (Fold1–Fold5) and mean AUC = 0.776 (95% CI: 0.625–0.927). (B) ROC curves from an independent validation set (30% hold-out), demonstrating model generalizability with mean AUC = 0.889 (95% CI: 0.739–1.000).

The black solid line represents the mean ROC curve across all folds or the final model performance; colored dashed lines represent individual fold results. AUC values > 0.7 indicate acceptable diagnostic accuracy, with values approaching 1.0 reflecting excellent performance. All analyses were performed using R software with the pROC package.
